# Analytical Model for the Tidal Evolution of the Evection Resonance and the Timing of Resonance Escape

**DOI:** 10.1029/2019je006266

**Published:** 2020-04-30

**Authors:** William R. Ward, Robin M. Canup, Raluca Rufu

**Affiliations:** 1 Planetary Science Directorate, Southwest Research Institute, Boulder, CO, USA

## Abstract

A high-angular momentum giant impact with the Earth can produce a Moon with a silicate isotopic composition nearly identical to that of Earth’s mantle, consistent with observations of terrestrial and lunar rocks. However, such an event requires subsequent angular momentum removal for consistency with the current Earth-Moon system. The early Moon may have been captured into the evection resonance, occurring when the lunar perigee precession period equals 1 year. It has been proposed that after a high- angular momentum giant impact, evection removed the angular momentum excess from the Earth-Moon pair and transferred it to Earth’s orbit about the Sun. However, prior N-body integrations suggest this result depends on the tidal model and chosen tidal parameters. Here, we examine the Moon’s encounter with evection using a complementary analytic description and the Mignard tidal model. While the Moon is in resonance, the lunar longitude of perigee librates, and if tidal evolution excites the libration amplitude sufficiently, escape from resonance occurs. The angular momentum drain produced by formal evection depends on how long the resonance is maintained. We estimate that resonant escape occurs early, leading to only a small reduction (~ few to 10%) in the Earth-Moon system angular momentum. Moon formation from a high-angular momentum impact would then require other angular momentum removal mechanisms beyond standard libration in evection, as have been suggested previously.

## Plain Language Summary

A canonical giant impact with the Earth by a Mars-sized impactor can produce the Moon and the current Earth-Moon angular momentum. However, such an impact would produce a planet and protolunar disk with very different proportions of impactor-derived material, likely leading to Earth-Moon compositional differences that are inconsistent with observed Earth-Moon isotopic similarities. Alternatively, a high-angular momentum impact could form a disk with a silicate composition similar to that of the Earth, but with a postimpact angular momentum much higher than in the current Earth-Moon system. As the early Moon tidally receded from the Earth, its perigee precession period lengthened. When this period equaled 1 year, the Moon may have been captured into the evection resonance with the Sun. It has been proposed that evection removed the angular momentum excess from the Earth- Moon pair, but the appropriate degree of angular momentum removal appears sensitive to tidal models. In this work, we use an analytical model to examine the Moon’s evolution in evection and find that escape from formal resonance occurs early, with limited angular momentum reduction. Thus, in order for a high-angular momentum giant impact to be consistent with the current Earth-Moon system, additional mechanisms that do not involve standard resonance occupancy appear required.

## 1. Introduction

The leading theory for lunar origin proposes that the Moon formed from material ejected into circumterrestrial orbit by a Mars-sized impactor colliding obliquely with the early Earth (Cameron & Ward, 1976). The impact theory became favored primarily for its ability to account for the Moon’s depletion in iron and the angular momentum (AM) of the current Earth-Moon system, *L*_EM_ = 3.5 × 10^41^ g cm^2^ s^−2^, with the latter implying an Earth day of about 5 hr when the Moon formed close to the Earth.

In what is sometimes referred to as the “canonical” case, a low-velocity, oblique impact of a Mars-sized body produces an Earth-disk system with an AM close to *L*_EM_ (e.g., Canup, 2004a, 2004b, 2008; Canup & Asphaug, 2001). Disks produced by canonical impacts are derived primarily from material originating in the impactor’s mantle. The isotopic composition of the impactor would have likely differed from that of the Earth (Kruijer & Kleine, 2017; Melosh, 2014; Pahlevan & Stevenson, 2007). Thus, a disk derived from the impactor would nominally yield a Moon whose composition differed from that of the Earth’s mantle.

Instead, the Moon and the silicate Earth have essentially identical isotopic compositions across all nonvolatile elements, including oxygen, chromium, titanium, silicon, and tungsten (e.g., Armytage et al., 2012; Kruijer et al., 2015; Lugmair & Shukolyukov, 1998; Touboul et al., 2007; Touboul et al., 2015; Wiechert et al., 2001; Zhang et al., 2012). Thus, a canonical impact appears to either require a low-probability compositional match between the impactor and Earth (e.g., Kruijer & Kleine, 2017), or that the disk and the postimpact Earth mixed and compositionally equilibrated after the impact but before the Moon formed (Lock et al., 2018; Pahlevan & Stevenson, 2007).

Alternatively, certain types of high AM impacts can directly produce a protolunar disk whose silicate composition is essentially identical to that of the Earth’s mantle (Canup, 2012; Ćuk & Stewart, 2012), accounting for nearly all Earth-Moon isotopic similarities without requiring an Earth-like impactor. Recent works on high-AM impacts in general suggest that due to the high energy of such events, the collisional aftermath can consist of a hot, pressure supported planet rotating uniformly out to its corotation limit, while beyond that the structure progressively transitions to a disk with a Keplerian profile (Lock et al., 2018; Lock & Stewart, 2017). It is argued that such structures, termed “synestias,” may have intermittently existed during the planetary accretion process and would have facilitated the formation of moons with compositions similar to that of their host planet via mixing and equilibration. However, all high-AM impacts leave the Earth-Moon system with a substantial AM excess compared to its current value, so that relevancy to lunar origin requires a reliable mechanism(s) to subsequently reduce the system AM.

Tidal interactions between the Earth and Moon conserve AM, but other processes can remove AM from the pair. Consider an initial lunar orbit that lies within the Earth’s equatorial plane and a lunar spin axis normal to that plane. The energy, *E,* and scalar AM, *L,* of the Earth-Moon system are

(1.1) $$E=\frac{1}{2}Cs^{2}+\frac{1}{2}C_{m}s_{m}^{2}-\frac{GMm}{2a},$$

(1.2) $$L=Cs+C_{m}s_{m}+m{\left(GMa\right)}^{1/2}{\left(1-e^{2}\right)}^{1/2}.$$

where *M* = 5.97 × 10^27^ g and *m* = 7.34 × 10^25^ g are the Earth and lunar masses, (*C*, *s*) and (*C*_*m*_, *s*_*m*_) are their principal moments of inertia and spin rates, respectively, and the final terms are the energy, *E*_*orb*_, and AM, *L*_*orb*_, of the lunar orbit, with *a* and *e* being its semi-major axis and eccentricity (see Table 1 for variable definitions). Over the age of the solar system, *L* has decreased due to a slowdown in the Earth’s spin caused by direct solar tides. Additionally, late-veneer impacts could stochastically change the AM of the Earth-Moon system (Bottke et al., 2010), and/or escaping material can remove AM as the Moon accretes (e.g., Kokubo et al., 2000; Salmon & Canup, 2012). However, these processes are thought to induce only small changes (by a few to 10%) insufficient to reconcile a high-AM impact with the current Earth-Moon.

The solar influence on the Earth-Moon system is not necessarily limited to its tides, and another AM removal process involves a resonance with the Sun. As the early Moon’s orbit expands due to tidal interaction with the Earth, it can be captured into the evection resonance, which occurs when the precession frequency of the Moon’s perigee, $\dot{\varpi }$ , equals that of the Earth’s solar orbit, Ω_⊙_ = 1.99 × 10^−7^s^−1^ (e.g., Brouwer & Clemence, 1961; Kaula & Yoder, 1976; Touma & Wisdom, 1998). Capture into evection excites the Moon’s orbital eccentricity and drains AM from the Earth-Moon pair, transferring it to Earth’s heliocentric orbit. Once the lunar eccentricity becomes sufficiently high, there is a phase during which the lunar orbit temporarily contracts due to the effects of lunar tides. Touma and Wisdom (1998) modeled capture of the Moon in evection for an initial terrestrial rotation period of 5 hr, assuming a lunar rotation synchronous with its mean motion and the Mignard tidal model, in which the tidal distortion forms some fixed time after the tide raising potential (see section 4.1). In their simulations, the Moon’s residence in evection is brief, with escape occurring soon after the lunar semi-major axis begins to contract, leading to only minor AM modification by a few percent of *L*_EM_ (e.g., Canup, 2008).

A key development was the work of Ćuk and Stewart (2012, hereafter CS12), who argued that if the original magnitude of *L* was substantially greater than at present, prolonged capture in evection could have reduced the Earth-Moon system AM by a factor of two or more. This would make it viable for a high-AM impact to have produced the Moon. CS12 utilized an ersatz tidal model intended to approximate a constant lag angle/constant-*Q* model, and considered an initial terrestrial day of only 2 to 3 hr, which shifts the position of evection outward in orbital radius relative to the cases in Touma and Wisdom (1998). The CS12 simulations showed a protracted residence of the Moon in evection that persisted even as the Moon’s orbit contracted and that during the contraction phase, large-scale AM removal comparable in magnitude to *L*_EM_ occurred. In their simulations, the final system AM when the Moon escapes from resonance depends on the relative strength of tidal dissipation in the Moon compared with that in the Earth, with the final AM achieving a minimum value close to *L*_EM_ across a relatively narrow range of this ratio. Their results thus implied that for certain tidal parameters, a final AM ≈ *L*_EM_ would be the limiting postevection system value, independent of the starting AM.

Considering the importance of this issue to the origin of the Moon and that AM removal due to evection depends on the Moon’s tidal evolution, it is imperative to understand the robustness of AM removal for other tidal models. Wisdom and Tian (2015) demonstrated that substantial differences in the AM removed compared with CS12 occur when a full constant-*Q* Darwin-Kaula model is applied (Kaula, 1964). They instead identified a “limit cycle” in which the system circulates around the stationary points associated with evection and appropriate AM can be lost even though the evection resonance angle is not librating, although this again appeared to require a relatively narrow range of tidal parameters (Tian et al., 2017; Wisdom & Tian, 2015). Further work that included the effects of tidal heating within an eccentrically orbiting Moon on the lunar tidal dissipation properties concluded that the evection resonance proper does not remove substantial AM, but that the limit cycle can (Tian et al., 2017).

In this paper, we examine the Moon’s evolution in evection using the Mignard tidal model as in Touma and Wisdom (1998), but we apply it to the higher AM systems considered in CS12 and consider the Moon’s potentially nonsynchronous rotation. All common tidal models have approximations and uncertainties. A strength of the Mignard model is its straightforward analytic form, whose tidal acceleration varies smoothly near synchronous orbit and for highly eccentric orbits in a physically intuitive manner. For the postgiant impact, fluid-like Earth (e.g., Zahnle et al., 2015), it seems reasonable that the position of the terrestrial equilibrium tide would reflect a characteristic time for the tide to form in the presence of internal dissipation, as assumed in the Mignard model, rather than a characteristic fixed angle relative to the Moon’s position, as assumed in the constant-*Q* tidal model. However, it is also the case that for the current Moon, the lunar tidal *Q* value does not display the inverse frequency dependence consistent with a time delay model, but instead varies weakly with frequency (e.g., Williams et al., 2014; Wisdom & Tian, 2015). In any case, the Mignard model permits a detailed examination of Earth-Moon-Sun interactions during the tidal evolution of the evection resonance to test whether the behavior first described in CS12 occurs with this model as well, and in so doing, to better understand the likelihood of large-scale modification of the Earth-Moon system AM.

## 2. Evection

We assume the Moon forms interior to the evection resonance on a low eccentricity orbit and then tidally evolves outward until it reaches the resonance site, *a*_*res*_, where the lunar apsidal precession rate equals the frequency of the Earth’s orbit. Because the lunar precession rate is a function of the Earth’s oblateness, which is in turn a function of Earth’s spin rate, the resonance location depends on Earth’s spin rate when the Moon formed. For an initial Earth-Moon AM, *L*_*o*_, equal to that in the current Earth-Moon system (*L*_EM_), evection is first encountered at *a*_*res*_ ~ 4.6*R*, where *R* is the Earth’s radius (e.g., Touma & Wisdom, 1998). An initial high-AM system with *L*_*o*_ ~ 2*L*_EM_ leads to *a*_*res*_ ~ 7*R* (e.g., CS12).

### 2.1. Lagrange Equations

We consider Earth on a circular orbit with zero obliquity and that the initial lunar inclination is negligible, so that the terrestrial and lunar orbits are coplanar. The disturbing function of the Sun acting on the Moon up to the second order Legendre polynomial and including only the oscillating term due to evection is (e.g., Brouwer & Clemence, 1961; Frouard et al., 2010)

(2.1) $$\Phi_{⊙}=-{\left(a\Omega_{⊙}\right)}^{2}\left[\frac{1}{4}+\frac{3}{8}e^{2}+\frac{15}{8}e^{2}\text{cos}2\phi \right],$$

where *ϕ* ≡ *ϖ* − *λ*_⊙_ is the resonance phase angle,*ϖ* is the Moon’s longitude of perigee, and *λ*_⊙_ is the solar longitude. The secular part of the potential for the Earth’s quadrupole field is (e.g., Efroimsky, 2005),

(2.2) $$\Phi_{⊕}=-\frac{1GMJ_{2}{\left(R/a\right)}^{2}}{2\;a\;{\left(1-e^{2}\right)}^{3/2}},$$

where *J*_2_ is the second order gravity coefficient. From Lagrange’s equations,

(2.3) $$\frac{de}{dt}=\frac{{\left(1-e^{2}\right)}^{1/2}}{na^{2}e}\frac{\partial \Phi_{⊙}}{\partial \varpi }=\frac{15}{4}e{\left(1-e^{2}\right)}^{1/2}\Omega_{⊙}\left(\frac{\Omega_{⊙}}{n}\right)\text{sin}2\phi ,$$

(2.4) $$\frac{d\varpi }{dt}=-\frac{{\left(1-e^{2}\right)}^{1/2}}{na^{2}e}\frac{\partial }{\partial e}\left(\Phi_{⊕}+\Phi_{⊙}\right)=\frac{3}{2}n\frac{J_{2}{\left(R/a\right)}^{2}}{{\left(1-e^{2}\right)}^{2}}+\frac{3}{4}{\left(1-e^{2}\right)}^{1/2}\Omega_{⊙}\left(\frac{\Omega_{⊙}}{n}\right)(1+5\text{cos}2\phi ),$$

where $n=\sqrt{GM/a^{3}}$ is the lunar mean motion. The apsidal precession rate is dominated by the Earth’s quadrupole and increases with the lunar eccentricity. In the vicinity of evection, $\dot{\varpi }$ approaches Ω_⊙_ and the phase angle, *ϕ*, changes slowly. The potential is stationary in a reference frame rotating with the Sun, so that in the absence of tides, an integral of the motion is given by the Jacobi constant (Appendix A in the supporting information),

(2.5) $$J=E_{\text{orb}}+\Phi_{⊕}+\Phi_{⊙}-\Omega_{⊙}L_{\text{orb}}=m\left[-GM/2a+\Phi_{⊕}+\Phi_{⊙}-\Omega_{⊙}\sqrt{GMa\left(1-e^{2}\right)}\right].$$

### 2.2. Normalized Forms

We normalize energy to $MR^{2}\Omega_{⊕}^{2}$, AM to *C*Ω_⊕_, where Ω_⊕_ ≡ (*GM*/*R*^3^)^1/2^ = 1.24 × 10^−3^*s*^−1^ is the orbital frequency at the surface of the Earth, and the semimajor axis to Earth radii. In these units, a scaled Earth spin AM of unity corresponds to rotation at approximately the stability limit. Equations (1.1) and (1.2) become

(2.6) $$E^{\prime }=\frac{\lambda }{2}{s^{\prime }}^{2}+\kappa \frac{\lambda }{2}{s^{\prime }}_{m}^{2}-\frac{\mu }{2a^{\prime }},$$

(2.7) $$L^{\prime }=s^{\prime }+\kappa s_{m}^{\prime }+\gamma {a^{\prime }}^{1/2}{\left(1-e^{2}\right)}^{1/2},$$

where s′ ≡ s/Ω⊕, $s_{m}^{\prime }\equiv s_{m}/\Omega_{⊕}$, and *a*′ ≡ *a*/*R*. Here *γ* ≡ *μ*/*λ* = 0.0367, *μ* ≡ *m*/*M*= 0.0123 is the Moon-Earth mass ratio, and *λ* ≡ *C*/*MR*^2^ = 0.335 is Earth’s gyration constant. The quantity *κ* ≡ *C*_*m*_/*C* = 1.07 × 10^−3^ is the ratio of maximum principal moments of inertia of the two bodies, while the final term of (2.7) is the normalized orbital AM of the Moon, $L_{\text{orb}}^{\prime }\equiv \gamma {a^{\prime }}^{1/2}{\left(1-e^{2}\right)}^{1/2}$. The equations for *ė* and $\dot{\phi }=\dot{\varpi }-\Omega_{⊙}$ take the nondimensional forms

(2.8) $$\frac{de}{d\tau }=\frac{15}{4}\chi e{\left(1-e^{2}\right)}^{1/2}{a^{\prime }}^{3/2}\left(\frac{\Omega_{⊙}}{\Omega_{⊕}}\right)\text{sin}2\phi ,$$

(2.9) $$\frac{d\phi }{d\tau }=\chi \left[\frac{\Lambda^{2}{s^{\prime }}^{2}}{{a^{\prime }}^{7/2}{\left(1-e^{2}\right)}^{2}}-1+\frac{3}{4}{\left(1-e^{2}\right)}^{1/2}{a^{\prime }}^{3/2}\left(\frac{\Omega_{⊙}}{\Omega_{⊕}}\right)(1+5\text{cos}2\phi )\right].$$

Here, we set *J*_2_= J_*_ *s*′^2^ to approximate the effect of the Earth’s spin on its oblateness, defined Λ ≡ [(3/2) J_*_Ω_⊕_/Ω_⊙_]^1/2^ and *χ* ≡ Ω_⊙_*t*_*T*_ and introduced a normalized time, *τ* ≡ *t*/*t*_*T*_, referenced to a tidal timescale, *t*_*T*_, that will depend on the tidal model. Numerical values are Ω_⊙_/Ω_⊕_ = 1.61 × 10^−4^, J_*_ = 0.315, and Λ = 54.2.

The Jacobi constant can also be written in a nondimensional form. The solar terms alter *e* and ϕ but do not change the energy of the Earth-Moon system, so that the Moon’s semimajor axis and the spin of the Earth are constants in the absence of tides. We scale *J* by $mR^{2}\Omega_{⊕}^{2}$, and then rearrange terms that do not depend on *e* or ϕ to define $J^{\prime }\equiv -\left(J/mR^{2}\Omega_{⊕}^{2}+1/2a^{\prime }\right)\left(\Omega_{⊕}/\Omega_{⊙}\right)/{a^{\prime }}^{1/2}-\left(\Omega_{⊙}/\Omega_{⊕}\right){a^{\prime }}^{3/2}/4$, which will be a constant in the absence of tides. This constant is given by

(2.10) $$J^{\prime }=\frac{1}{3}\frac{\Lambda^{2}{s^{\prime }}^{2}}{{a^{\prime }}^{7/2}{\left(1-e^{2}\right)}^{3/2}}+{\left(1-e^{2}\right)}^{1/2}+\frac{3\Omega_{⊙}}{8\Omega_{⊕}}{a^{\prime }}^{3/2}e^{2}(1+5\text{cos}2\phi ).$$

Since the equations of motion depend on the square of the eccentricity, we introduce the variable *ε* = *e*^2^, as well as the angle, *θ* ≡ *ϕ* − *π*/2, which is the libration angle relative to the positive *y*-axis in the direction of the negative *x*-axis (with the Sun positioned along the positive *x*-axis), so that *θ* = 0, *π* correspond to the stable points for evection (see below). Finally, we define the quantities.

(2.11) $$\eta \equiv \Lambda s^{\prime }/{a^{\prime }}^{7/4};\alpha \equiv \alpha_{0}{a^{\prime }}^{3/2};\alpha_{o}\equiv (3/8)\Omega_{⊙}/\Omega_{⊕}$$

with *α*_*o*_ = 6.04 × 10^−5^. The evolution equations due to evection and the related Jacobi constant simplify to

(2.12) $$\dot{\varepsilon }=-20\chi \alpha \varepsilon {\left(1-\varepsilon \right)}^{1/2}\text{sin}2\theta ,$$

(2.13) $$\dot{\theta }=\chi \left[\eta^{2}/{\left(1-\varepsilon \right)}^{2}-1+2\alpha {\left(1-\varepsilon \right)}^{1/2}(1-5\text{cos}2\theta )\right],$$

(2.14) $$J^{\prime }=\eta^{2}/\left[3{\left(1-\varepsilon \right)}^{3/2}\right]+{\left(1-\varepsilon \right)}^{1/2}+\alpha \varepsilon (1-5\text{cos}2\theta ).$$

### 2.3. Stationary States

Stationary points of the resonance occur where $\dot{\varepsilon }=\dot{\theta }=0$. The value $\dot{\varepsilon }$ vanishes at *θ* = 0, *π*/2, *π*, and 3*π*/2, while $\dot{\theta }=0$ occurs when

(2.15) $$(1-\varepsilon ){\left[1-2\alpha (1-5\text{cos}2\theta ){\left(1-\varepsilon \right)}^{1/2}\right]}^{1/2}=\eta .$$

When the resonance is fully developed, there are four stationary points at (*ε*, *θ*) = (*ε*_*s*_,0), (*ε*_*s*_,*π*) and (*ε*_*sx*_, ± *π*/2). Finally, *ε* = 0 is also a stationary point since it implies $\dot{\varepsilon }=0$, although in this case, the angle *θ* is degenerate.

### 2.4. Expansion to $O\left(e^{4}\right)$

We now expand the governing expressions to $O\left(e^{4}\right)=O\left(\varepsilon^{2}\right)$, a reasonable approximation for *ε* < 0.4 (i.e., *e* ≤ 0.6) that provides sufficient accuracy to capture the relevant behavior (e.g., Murray & Dermott, 1999; Touma & Wisdom, 1998). The variable *α* is small, of order few × 10^−4^ to 5 × 10^−3^ for 3 < *a*′ < 20. Expanding equation (2.15) to lowest order in α gives *ε* ≈ 1 − *η* − *α*(1 − 5cos2*θ*)*η*^3/2^, and by further neglecting O(αε) terms (that will typically be smaller than *O*(*ε*^2^) terms), we find approximate expressions for the stationary points (Appendix B in the supporting information),

(2.16) $$\varepsilon_{s}=\varepsilon_{*}+5\alpha ;\varepsilon_{sx}=\varepsilon_{*}-5\alpha ,$$

whose average is

(2.17) $$\varepsilon_{*}\approx 1-\eta -\alpha ,$$

which in turn implies $1-\eta^{2}=1-{\left[\left(1-\varepsilon_{*}\right)-\alpha \right]}^{2}\approx 2(1-\eta )-\varepsilon_{*}^{2}$. Expanding equation (2.14) and rearranging gives

(2.18) $$J^{\prime }-1-\eta^{2}/3=\left(5\eta^{2}-1\right)\varepsilon^{2}/8-\left(1-\eta^{2}-2\alpha +10\alpha \text{cos}2\theta \right)\varepsilon /2\equiv \tilde{J}.$$

Consistent with $O\left(\varepsilon^{2}\right)$ accuracy, we further simplify (2.18) by setting *η* → 1 in the coefficient of *ε*^2^, 1 − *η*^2^ ≈ 2(1 − *η*) in the *ε* coefficient, and defining

(2.19) $$\beta \equiv 1-\eta -\alpha (1-5\text{cos}2\theta )\approx \varepsilon_{*}+5\alpha \text{cos}2\theta ,$$

so that

(2.20a,b) $$\tilde{J}\approx (\varepsilon -2\beta )\varepsilon /2;\;\varepsilon =\beta \pm \sqrt{\beta^{2}+2\tilde{J}},$$

where the first expression gives the Jacobi constant to $O\left(\varepsilon^{2}\right)$, and the second gives the solutions for *ε*(*θ*) from this quadratic equation. The rates of change for the eccentricity and resonance angle that are compatible with this approximation become.

(2.21a,b) $$\dot{\varepsilon }=-20\chi \alpha \varepsilon \text{sin}2\theta ;\dot{\theta }=2\chi \left(\varepsilon -\varepsilon_{*}-5\alpha \text{cos}2\theta \right)=2\chi (\varepsilon -\beta )$$

where in the last expression, we have dropped a term $\chi \left(\varepsilon_{*}^{2}-\varepsilon \left(4\varepsilon_{*}-3\varepsilon \right)\right)$; because for an eccentricity similar to that of the stationary point, i.e., *ε* ~ ε_*_ ± 5*α*, $\left(\varepsilon_{*}^{2}-\varepsilon \left(4\varepsilon_{*}-3\varepsilon \right)\right)$ is O(αε). We utilize equations (2.21a,b) to describe the effects of evection on the system evolution in sections 5 and 6.

## 3. Evection Level Curves

Given a terrestrial spin rate and lunar semimajor axis (which define *η* and *α*), the Jacobi constant defines the set of allowed (*ε*, *θ*) combinations. Using the $O\left(\varepsilon^{2}\right)$ expressions in equations (2.19) and (2.20), we set

(3.1) $$\frac{\varepsilon }{5\alpha }=\left[\frac{\varepsilon_{*}}{5\alpha }+\text{cos}2\theta \right]\pm {\left[{\left(\frac{\varepsilon_{*}}{5\alpha }+\text{cos}2\theta \right)}^{2}+\frac{2\tilde{J}}{{\left(5\alpha \right)}^{2}}\right]}^{1/2}.$$

**FIGURE 1.**
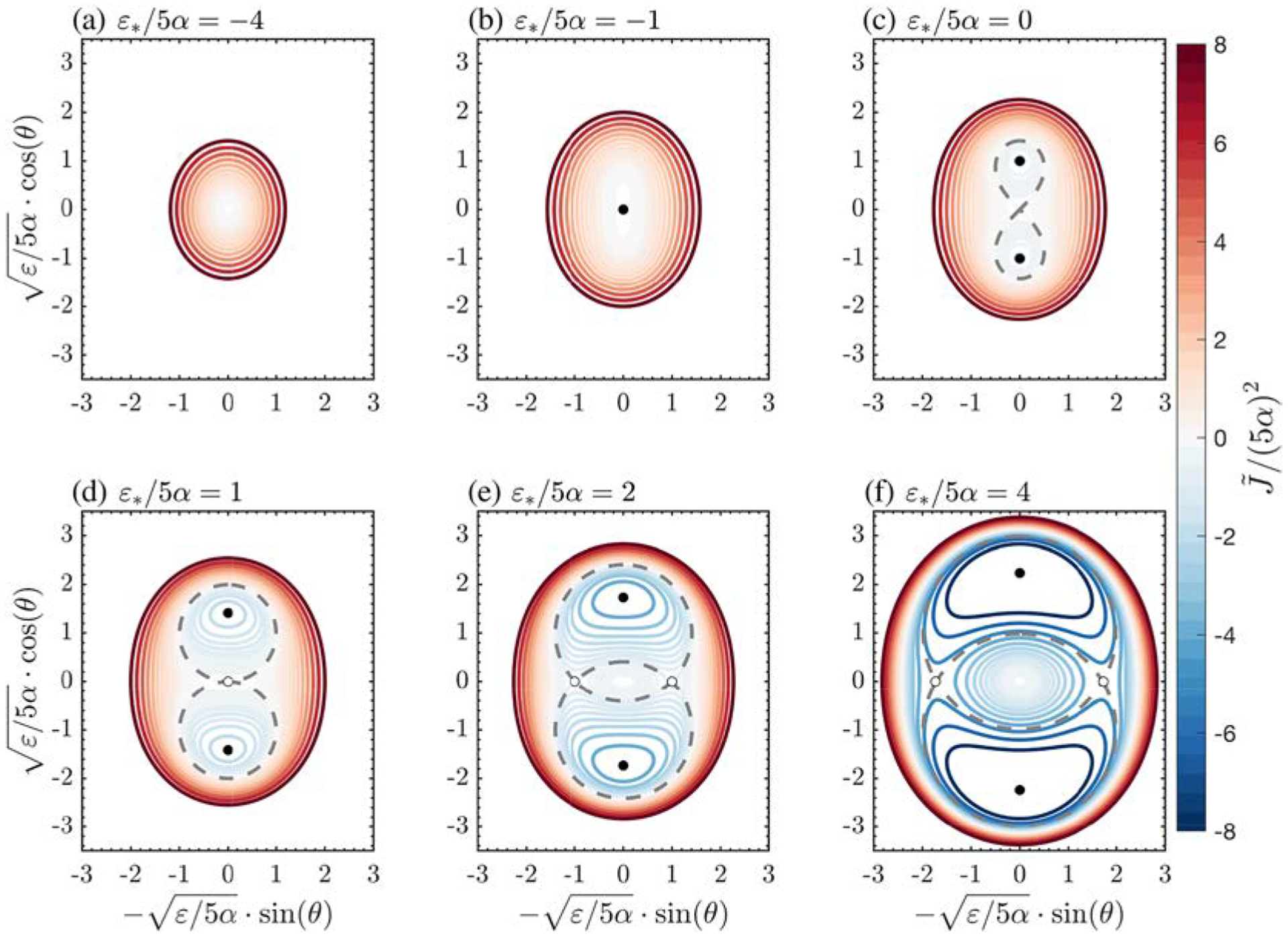
Level curves for the evection resonance for different energies. The Sun is in the direction of the positive *x*-axis. (a) ε_*_/5*α* = − 4: Pre-resonance where all motion is counter-clockwise circulation about the origin. (b) ε_*_/5*α* = − 1: First appearance of stable stationary states on *y*-axis. (c) ε_*_/5*α* = 0: Shallow resonance where the level curve $\tilde{J}=0$ is a separatrix dividing counter-clockwise libration about the stationary point from level curves circulating the origin. (d) ε_*_/5*α* = 1: First appearance of unstable saddle points on the *x*-axis. (e) ε_*_/5*α* = 2: Deep resonance with a separatrix composed of two branches emanating from the saddle points. Below the lower branch are level curves circulating the origin in the clockwise direction. (f) ε_*_/5*α* = 4: Still further into deep resonance, with the saddle points farther apart.

Figure 1 shows the resulting level curves with $x=-\sqrt{\varepsilon /5\alpha }\text{sin}\theta$ and $y=\sqrt{\varepsilon /5\alpha }\text{cos}\theta$ for constant $\tilde{J}/{\left(5\alpha \right)}^{2}$ values for several ε_*_/5*α* values. The radial distance from the origin is equal to $\sqrt{\varepsilon /5\alpha }$ (and thus proportional to *e*), while *θ* is the angle from the *y*-axis in the direction of the negative *x-*axis.

The external solar torque is found from equation (2.1) with T = − *m*∂Φ_⊙_/∂*θ*, viz.,

(3.2a,b) $$\text{T}=(15/4)m{\left(a\Omega_{⊙}\right)}^{2}\varepsilon \text{sin}2\theta ;\text{T}\prime =10\gamma \chi \alpha {a^{\prime }}^{1/2}\varepsilon \text{sin}2\theta$$

the latter being its normalized version, i.e., T′ ≡ T/(*C*Ω_⊕_/*t*_*T*_) = T/(*C*Ω_⊕_Ω_⊙_/*χ*). All level curves in Figure 1 have reflection symmetry across the *y*-axis, and the value of *ε* at *θ* = *θ*_0_ is equal to that at *θ* = − *θ*_0_. Thus, the solar torque at *θ* = *θ*_0_ will be of equal magnitude but opposite sign to that at *θ* = − *θ*_0_ due to the sin2*θ* term in (3.2), and the net solar torque (and thus the change in Earth-Moon AM) over a libration cycle is zero in the absence of tides.

### 3.1. Separatrix

At the stationary points $\dot{\theta }=0$, and so from (2.21b), *ε*_*s*_= *ε*_*sx*_ = *β*. Jacobi values at the stationary points are ${\tilde{J}}_{s}=-\varepsilon_{s}^{2}/2$ and ${\tilde{J}}_{sx}=-\varepsilon_{sx}^{2}/2$. The partial derivative of $\tilde{J}$ with respect to *ε* is simply $\partial \tilde{J}/\partial \varepsilon =\varepsilon -\beta$ and vanishes at the stationary points, while $\partial^{2}\tilde{J}/\partial \varepsilon^{2}=1$ is positive, indicating a relative minimum. On the other hand, while $\partial \tilde{J}/\partial \theta =10\alpha \varepsilon \;\text{sin}2\theta$ also vanishes, $\partial^{2}\tilde{J}/\partial \theta^{2}=20\alpha \varepsilon \;\text{cos}2\theta$ is positive on the *y*-axis (when *θ* = 0, *π*) but negative on the *x*-axis (when *θ* = ± *π*/2). Accordingly, on the *y*-axis, the stationary points are absolute minima and stable (*ε*_*s*_; Figure 1, filled markers), while on the *x*-axis, they are unstable saddle points (*ε*_*sx*_; Figure 1, open markers). Note that $\tilde{J}$ is always zero at the origin and (per equation 2.20a) along the trajectory *ε* = 2*β*, provided that *β* > 0.

The level curve passing through the saddle points (Figure 1, dashed curve) is a separatrix that partitions resonant trajectories, which librate about the stable stationary points, from nonresonant trajectories that circulate around the origin. The value of *ε* along the separatrix can be found by setting $\tilde{J}={\tilde{J}}_{sx}$ in equation (2.20b) to give

(3.3) $$\varepsilon_{\pm }=\varepsilon_{*}+5\alpha \text{cos}2\theta \pm {\left[{\left(\varepsilon_{*}+5\alpha \text{cos}2\theta \right)}^{2}-\varepsilon_{\alpha }^{2}\right]}^{1/2}.$$

where *ε*_+_ (*ε*_−_) denotes the radially outer (radially inner) curve. The maximum and minimum *ε*_±_ occur at *θ* = 0, *π*/2:

(3.4) $$\varepsilon_{\pm }\to \varepsilon_{s}\pm {\left(\varepsilon_{s}^{2}-\varepsilon_{sx}^{2}\right)}^{1/2}=\varepsilon_{s}\pm 2\sqrt{5\alpha \varepsilon_{*}}.$$

### 3.2. Resonance Domains

As ε_*_/5*α* increases from initially negative (precapture) values to positive values, different domains emerge on the level curve diagrams. Let ϒ_1_ refer to the domain area where the level curves circulate the origin in the counter-clockwise direction. When ε_*_/5*α* < − 1, this is the only domain that exists (Figure 1a). This is precapture behavior where $\tilde{J}$ must be positive because *β* < 0 in this domain. In this stage both *ε*_*s*_ and *ε*_*sx*_ are negative and so *e*_*s*_ and *e*_*sx*_ are undefined.

A smaller *s*′ and/or larger *a*′ increases ε_*_. When −1 < ε_*_/5*α* < 1, the stable stationary points *ε*_*s*_ first appear at the origin (Figure 1b). With increasing ε_*_, the stationary points *ε*_*s*_ move outward along the *y*-axis (Figures 1c and 1d). The Jacobi constant can then take on negative values down to ${\tilde{J}}_{s}=-\varepsilon_{s}{}^{2}/2$, which is an absolute minimum, while the level curve for $\tilde{J}=0$, *viz*., *ε* = 2*β*, becomes a boundary that separates trajectories that still circulate the origin (in domain ϒ_1_) from a new class that librate about the stable stationary point within a new domain ϒ_2_. We refer to this initial stage in resonance in which only domains ϒ_1_ and ϒ_2_ exist as shallow resonance.

**FIGURE 2.**
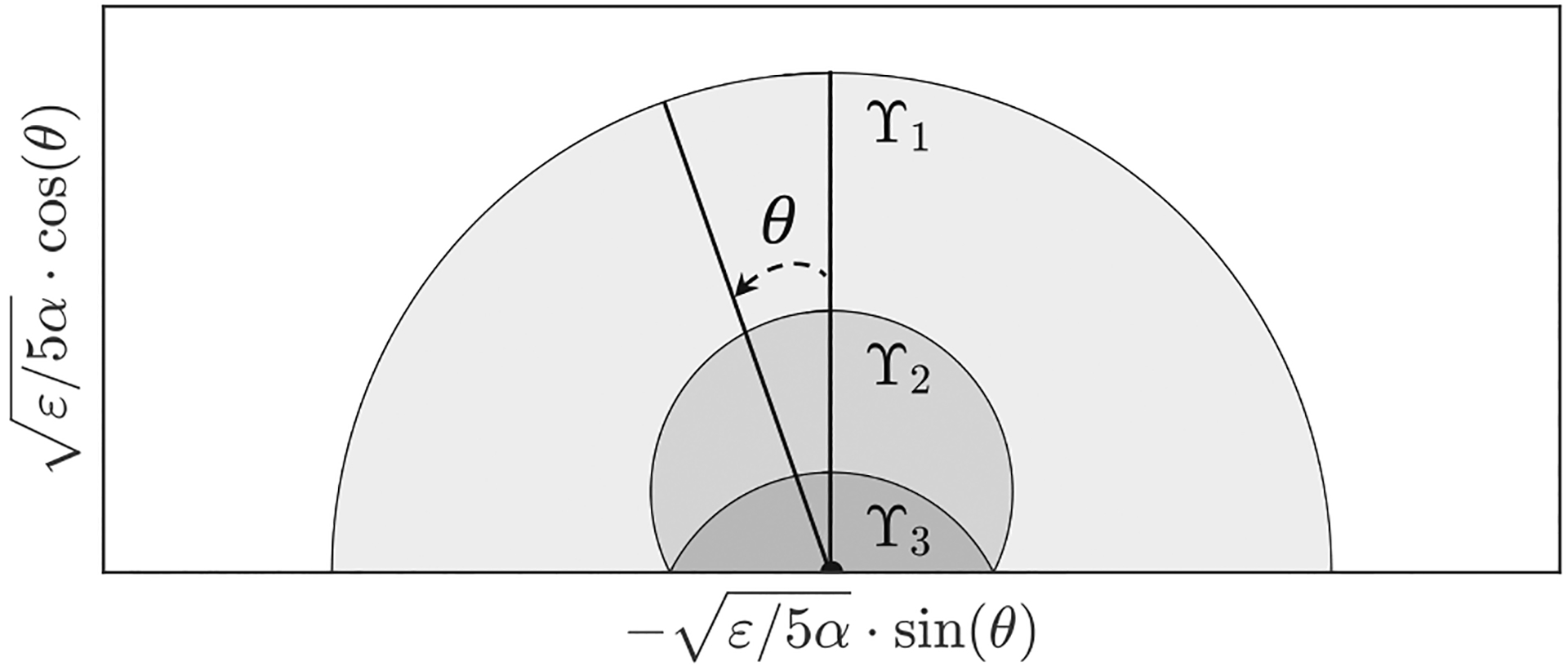
Schematic of level curve domains in deep resonance (location of outer ϒ_1_ domain not shown to scale).

For ε_*_/5*α* > 1, the minimum value of $\tilde{J}$ along the *x*-axis is no longer at the origin but occurs at new stationary points at (*ε*, *θ*) = (*ε*_*sx*_, −*π*/2) and (*ε*_*sx*_, *π*/2). These are the saddle points where the two branches of the ${\tilde{J}}_{sx}=-\varepsilon_{sx}^{2}/2$ curve connect. Trajectories for $\tilde{J}<{\tilde{J}}_{sx}$ still librate about the stationary points on the *y*-axis in domain ϒ_2_. For $\tilde{J}>{\tilde{J}}_{sx}$, trajectories beyond the outer separatrix boundary circulate the origin counterclockwise, but within the lower separatrix boundary, there is now a new, lens-shaped domain ϒ_3_, where nonresonant trajectories circulate the origin in a clockwise sense (Figures 1e–1f). We refer to this stage as deep resonance, whose structure above the *x*-axis is illustrated schematically in Figure 2.

## 4. Tidal Friction

The level curve patterns are set by the Earth’s spin rate and the lunar semimajor axis through ε_*_ and *α*, which evolve due to tidal friction between the Earth and Moon.

Earth-Moon tides exchange AM between the objects’ spins and the lunar orbit but do not change the total Earth-Moon AM. We represent the semimajor axis and eccentricity rates of change due to tides raised on the Earth by the Moon as ${\dot{a}}_{⊕}^{\prime }$ and ${\dot{\varepsilon }}_{⊕}$, while ${\dot{a}}_{m}^{\prime }$ and ${\dot{\varepsilon }}_{m}$ denote corresponding rates for tides raised on the Moon by the Earth. Tides alter the respective spins of the Earth and Moon at rates

(4.1) $${\dot{s}}^{\prime }=-(\gamma /2){a^{\prime }}^{1/2}{\left(1-\varepsilon \right)}^{1/2}[{{\dot{a}}^{\prime }}_{⊕}/a^{\prime }-{\dot{\varepsilon }}_{⊕}/(1-\varepsilon )],$$

(4.2) $${{\dot{s}}^{\prime }}_{m}=-(\gamma /2\kappa ){a^{\prime }}^{1/2}{\left(1-\varepsilon \right)}^{1/2}\left[{{\dot{a}}^{\prime }}_{m}/a^{\prime }-{\dot{\varepsilon }}_{m}/(1-\varepsilon )\right]$$

In these expressions, the time derivatives use the afore mentioned time variable *τ* = *t*/*t*_*T*_, to be specified below. Conservation requires that the change in the Moon’s orbital AM due to tides is ${{\dot{L}}^{\prime }}_{\text{orb},T}=-{\dot{s}}^{\prime }-\kappa {{\dot{s}}^{\prime }}_{m}$ or

(4.3) $${{\dot{L}}^{\prime }}_{\text{orb},T}={L^{\prime }}_{\text{orb}}\left[{\dot{a}}^{\prime }/a^{\prime }-{\dot{\varepsilon }}_{T}/(1-\varepsilon )\right]/2$$

where ${\dot{a}}^{\prime }={\dot{a}}_{⊕}^{\prime }+{{\dot{a}}^{\prime }}_{m}$ and ${\dot{\varepsilon }}_{T}={\dot{\varepsilon }}_{⊕}+{\dot{\varepsilon }}_{m}$ are the total rates of change from both Earth and lunar tides.

The equations of motion derived in section 2.4 (equation 2.21a,b) must be modified to include tidal changes, with

(4.4a,b) $$\dot{\varepsilon }=-20\chi \alpha (\tau )\varepsilon \text{sin}2\theta +{\dot{\varepsilon }}_{T};\dot{\theta }=2\chi \left[\varepsilon -\varepsilon_{*}(\tau )-5\alpha (\tau )\text{cos}2\theta \right].$$

As a result, a time independent first integral (Jacobi constant) no longer exists, with

(4.5) $$\dot{\tilde{J}}=\left(\varepsilon -\varepsilon_{*}-5\alpha \text{cos}2\theta \right){\dot{\varepsilon }}_{T}-\left({\dot{\varepsilon }}_{*}+5\dot{\alpha }\text{cos}2\theta \right)\varepsilon ,$$

and system trajectories on a level curve diagram are not closed. Since both ε_*_ and *α* vary with time, so do *ε*_*s*_ and *ε*_*sx*_, although on a tidal timescale much longer then a libration period, i.e., they are quasi-stationary states.

### 4.1. Mignard Tidal Model

We employ the model of Mignard (1980), in which the rise of the equilibrium tidal distortion is delayed by a fixed time relative to the tide raising stress. We define the tidal time constant, $t_{T}\equiv {\left(6k_{T}\mu \Omega_{⊕}^{2}\Delta t\right)}^{-1}$, where *k*_*T*_ is the Earth’s second degree tidal Love number and Δ*t* is the terrestrial time delay; for the current Earth, Δ*t* ≈ 12 min, and $t_{T}\sim 4\times 10^{-4}k_{T}^{-1}$ years. The constant time delay results in a frequency-dependent lag-angle between the tide and the line connecting the Earth-Moon centers, *δ* = (*s* − *n*)Δ*t*, where *δ* varies smoothly as frequencies approach and pass through the *s* = *n* case (i.e., a spin synchronous with the lunar mean motion). This is a key advantage of the Mignard model compared with the Darwin-Kaula constant lag-angle tidal model (e.g., Kaula, 1964), in which the lag angle has discontinuities near commensurabilities (e.g., Kaula, 1964; Tian et al., 2017).

#### 4.1.1. Earth Tides

Considering the second harmonic in the tidal potential, the Mignard equations for the evolution of *a*′ and *ε* vs. *τ* = *t*/*t*_*T*_ due to Earth tides are

(4.6a) $${\dot{a}}_{⊕}^{\prime }/a^{\prime }=(1+\mu )\left[s^{\prime }{a^{\prime }}^{3/2}f_{1}(\varepsilon )-f_{2}(\varepsilon )\right]/{a^{\prime }}^{8}$$

(4.6b) $${\dot{\varepsilon }}_{⊕}=(1+\mu )\varepsilon \left[s^{\prime }{a^{\prime }}^{3/2}g_{1}(\varepsilon )-g_{2}(\varepsilon )\right]/{a^{\prime }}^{8}$$

with *f*_1_, *f*_2_, *g*_1_ and *g*_2_ given by

(4.7a,b) $$f_{1}(\varepsilon )={\tilde{f}}_{1}(\varepsilon )/{\left(1-\varepsilon \right)}^{6};f_{2}(\varepsilon )={\tilde{f}}_{2}(\varepsilon )/{\left(1-\varepsilon \right)}^{15/2}.$$

(4.7c,d) $$g_{1}(\varepsilon )={\tilde{g}}_{1}(\varepsilon )/{\left(1-\varepsilon \right)}^{5};g_{2}(\varepsilon )={\tilde{g}}_{2}(\varepsilon )/{\left(1-\varepsilon \right)}^{13/2}.$$

where ${\tilde{f}}_{1}$, ${\tilde{f}}_{2}$, ${\tilde{g}}_{1}$, and ${\tilde{g}}_{2}$ are polynomials in *ε* (Table 2) found by orbit averaging the tidal forces. Combining these with equation (4.1), the de-spin rate of the Earth is

(4.8) $${\dot{s}}^{\prime }=-\frac{\gamma (1+\mu )}{2{a^{\prime }}^{15/2}}\left(s^{\prime }{a^{\prime }}^{3/2}\frac{{\tilde{f}}_{1}-\varepsilon {\tilde{g}}_{1}}{{\left(1-\varepsilon \right)}^{11/2}}-\frac{{\tilde{f}}_{2}-\varepsilon {\tilde{g}}_{2}}{{\left(1-\varepsilon \right)}^{7}}\right).$$

#### 4.1.2. Lunar Tides

The corresponding evolution expressions due to satellite tides are

(4.9a) $${{\dot{a}}^{\prime }}_{m}/a^{\prime }=(1+\mu )A\left[{s^{\prime }}_{m}{a^{\prime }}^{3/2}f_{1}(\varepsilon )-f_{2}(\varepsilon )\right]/{a^{\prime }}^{8},$$

(4.9b) $${\dot{\varepsilon }}_{\text{m}}=(1+\mu )A\varepsilon \left[{s^{\prime }}_{m}{a^{\prime }}^{3/2}g_{1}(\varepsilon )-g_{2}(\varepsilon )\right]/{a^{\prime }}^{8},$$

where

(4.10) $$A\equiv \left(\frac{k_{m}}{k_{T}}\right)\left(\frac{\Delta t_{m}}{\Delta t}\right){\left(\frac{M}{m}\right)}^{2}{\left(\frac{R_{m}}{R}\right)}^{5}\approx 10\left(\frac{k_{m}}{k_{T}}\right)\left(\frac{\Delta t_{m}}{\Delta t}\right)$$

is a ratio of physical parameters of the two bodies that scales the relative strength of tides on the Moon to tides on the Earth, with *R*_*m*_, *k*_*m*_, and Δ*t*_*m*_ referring to the Moon’s radius, tidal Love number and tidal time delay (Mignard, 1980). For the current Earth and Moon, *A* ≈ unity. However, when the early Moon encountered evection, the post-giant impact Earth would have still been fully molten, with a tidal response akin to that of a fluid body with a small Δ*t*, while the Moon would have likely cooled sufficiently to yield a dissipative state with a much larger Δ*t*_*m*_, implying *A* ≫ 1 when the Moon encountered the resonance (Zahnle et al., 2015).

Combining equations (4.9a), (4.9b), and (4.10) with equation (4.2), the change in the lunar spin rate is

(4.11) $${\dot{s}}_{m}^{\prime }=-\frac{\gamma }{2\kappa }\frac{A(1+\mu )}{a^{\prime 15/2}}\left[s_{m}^{\prime }{a^{\prime }}^{3/2}\frac{{\tilde{f}}_{1}-\varepsilon {\tilde{g}}_{1}}{{\left(1-\varepsilon \right)}^{11/2}}-\frac{{\tilde{f}}_{2}-\varepsilon {\tilde{g}}_{2}}{{\left(1-\varepsilon \right)}^{7}}\right]$$

Because the Moon-Earth mass ratio *μ* is small, we set (1+*μ*) ≈ 1 in all subsequent tidal rate expressions.

#### 4.1.3. Lunar Rotation

For a synchronously rotating satellite, *s*^′^_*m*_*a*′^3/2^= 1, and the above equations would simplify to ${{\dot{a}}^{\prime }}_{m}/a^{\prime }=A\left(f_{1}-f_{2}\right)/{a^{\prime }}^{8}$, ${\dot{\varepsilon }}_{m}=A\varepsilon \left(g_{1}-g_{2}\right)/{a^{\prime }}^{8}$, and ${\dot{s}}_{m}^{\prime }=0$. However, there is a contradiction here. For an eccentric orbit with *ε* > 0, equation (4.2) shows that the satellite’s spin will not remain at a constant value of *s*^′^_*m*_*a*′^−3/2^ if subject to tidal rates given in equation (4.9a,b). Instead there will be a nonzero torque on the satellite spin that will move it away from synchronicity until

(4.12) $${s^{\prime }}_{m}{a^{\prime }}^{3/2}=\frac{(1-\varepsilon )f_{2}-\varepsilon g_{2}}{(1-\varepsilon )f_{1}-\varepsilon g_{1}},$$

which is only unity for a circular orbit (*ε* = 0), implying nonsynchronous rotation for an eccentrically orbiting satellite. Synchronous rotation can be maintained in an eccentric orbit if an additional torque is exerted on a permanent triaxial figure of the Moon (e.g., Aharonson et al., 2012; Goldreich, 1966; Goldreich & Peale, 1966a, 1966b). The original Mignard equations that assumed synchronous rotation did not include this permanent figure torque. Appendix D in the supporting information develops expressions to include this torque’s effects on *a* and *ε* for cases in which synchronous lunar rotation is assumed.

### 4.2. Resonance Encounter

**FIGURE 3.**
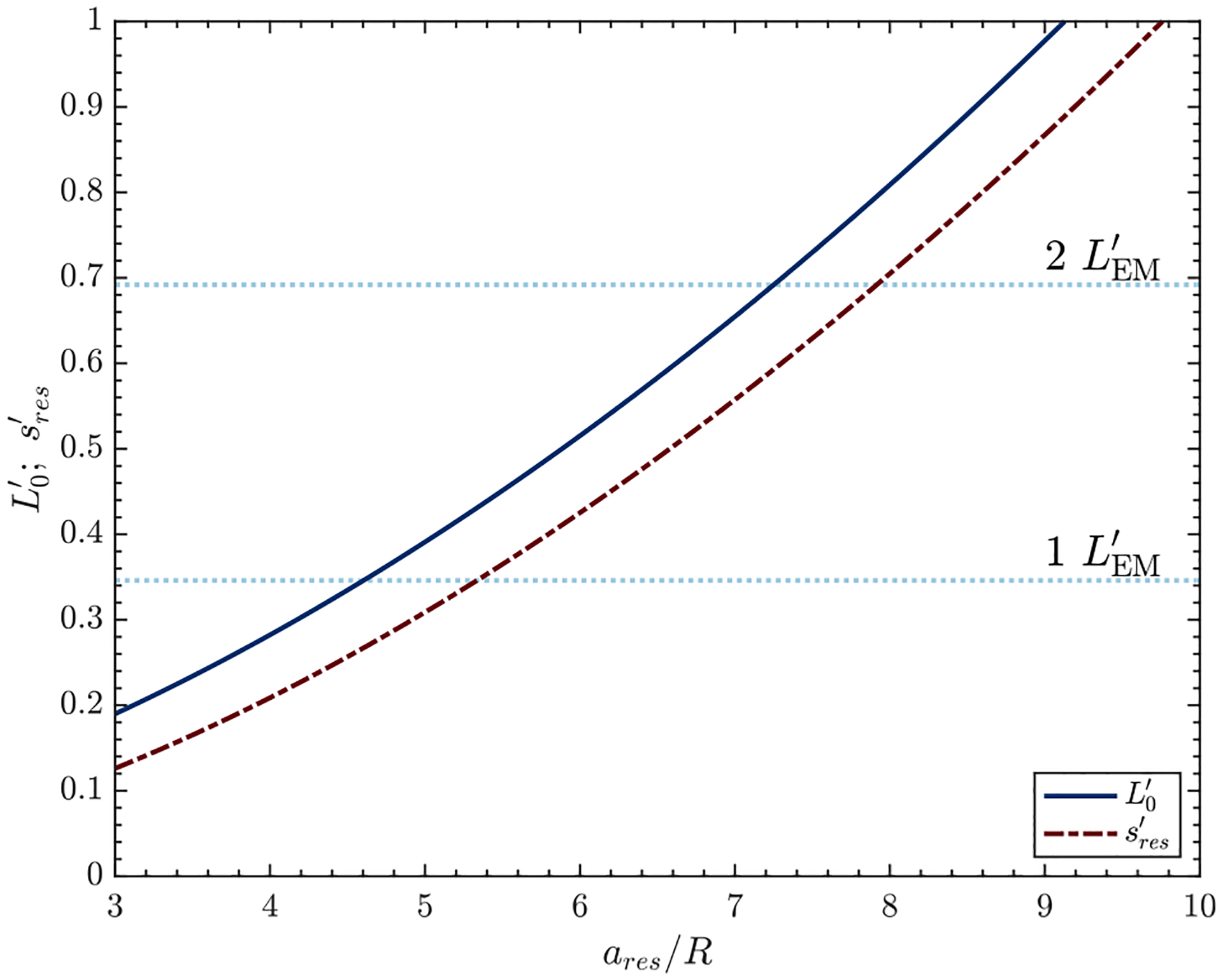
(a) The initial system angular momentum, *L*_*o*_, (assuming an initial near circular orbit of the Moon at three Earth radii) that would result in evection resonance location *a*_*res*_. Also shown (dashed curve) is the Earth spin, *s*′_*res*_, at resonance encounter. The current Earth-Moon angular momentum, *L*_*EM*_, as well as twice its value are shown for comparison (dotted lines).

During the initial precapture expansion of the lunar orbit, ε_*_ is negative but increasing. Shallow resonance is first established when ε_*_ =−5*α* and *s*′ = *a*′^7/4^(1+4*α*)/Λ. Assuming that prior to that the Moon’s orbit was circular, its spin synchronous, and the system AM, $L_{o}^{\prime }$, conserved, yields the constraint

(4.13) $${L^{\prime }}_{o}=\left(1+4\alpha_{o}{a^{\prime }}_{res}^{3/2}\right){a^{\prime }}_{res}^{7/4}/\Lambda +\kappa /{a^{\prime }}_{res}{}^{3/2}+\gamma {a^{\prime }}_{res}^{1/2}$$

for the resonance encounter distance as displayed in Figure 3. For $L_{o}^{\prime }$ equal to the current system value, $L_{\text{EM}}^{\prime }=0.346$, *a*′_*res*_ = 4.61 and *s*′_*res*_ = 0.267. For a high-AM state with *L*′_*o*_ ≈ 2*L*′_EM_, one obtains *a*′_*res*_ = 7.30 and *s*′_*res*_ = 0.596. For a low-AM state with $L_{o}^{\prime }<0.190\;\left(0.549L_{\text{EM}}\right)$, evection would lie interior to the Roche limit and would not be encountered as the Moon’s orbit tidally expanded.

Capture into resonance requires that tidally driven changes in the stationary point occur slowly compared to the resonant libration timescale. To understand the condition required to maintain the resonance, we differentiate equation (2.21b) (in the limit of no tides) and then use both (2.21a,b) to eliminate $\dot{\varepsilon }$ and $\dot{\theta }$ to yield,

(4.14) $$\ddot{\theta }=-40\chi^{2}\alpha \left(\varepsilon_{*}+5\alpha \text{cos}2\theta \right)\text{sin}2\theta \approx -80\chi^{2}\alpha \varepsilon_{s}\theta ,$$

where the final version assumes small *θ*. This is the equation for a harmonic oscillator of frequency $\omega =4\chi \sqrt{5\alpha \varepsilon_{s}}$ that is librating about a stable equilibrium point. The libration frequency increases with *ε*_*s*_. When in the shallow resonant regime, if the time it takes to execute a half cycle around the stationary point, ~*π*/*ω*, is comparable to or shorter than the time for that point to reach a given *ε*_*s*_ value viatides, $\sim \varepsilon_{s}/\left|{\dot{\varepsilon }}_{s}\right|$, capture into region ϒ_2_ can occur. This condition requires that $\varepsilon_{s}\geq {\left[\pi {\dot{\varepsilon }}_{s}/(4\chi \sqrt{5\alpha })\right]}^{2/3}$. For slow tides $\left(\text{ small }{\dot{\varepsilon }}_{s}\right)$, this can be satisfied for small *ε*_*s*_, but the needed *ε*_*s*_ value increases for faster tidal evolution. On the other hand, once *ε*_*s*_ ≥ 10*α* (i.e., once ε_*_/5*α* ≥ 1) the saddle points, *ε*_*sx*_, appear, and an increasing portion of phase space becomes occupied by the inner nonresonant region ϒ_3_ (e.g., Figure 2), causing the resonant region ϒ_2_ to radially narrow. This makes the adiabatic condition for resonance stability more stringent as ε_*_/5*α* increases further, because smaller tidally driven changes in *ε*_*s*_ during a libration cycle can cause the trajectory to pass directly from ϒ_1_ to ϒ_3_, avoiding resonance capture.

## 5. Evolution: Damped Libration

We first construct a baseline evolutionary track by restricting the Moon’s resonance behavior to one of zero libration amplitude, for which the eccentricity equals that of the stable stationary state (i.e., we set *ε* = *ε*_*s*_, *θ* = 0, and ignore equations (4.4a,b) associated with libration about the stationary state). While obviously idealized, the damped libration solution reveals how AM drain occurs and when in the evolution it would be most significant if the resonance is maintained. In section 6, we expand on this baseline evolution to estimate when libration amplitude growth and resonance escape is expected.

It is uncertain whether the Moon would have had a permanent triaxial moment when it encountered evection. Our nominal damped libration cases consider a nonsynchronously rotating moon without a permanent figure. An example case assuming a triaxial moon in synchronous rotation is presented in Appendix E in the supporting information (Figure SA3). For nonsynchronous cases, we assume that the lunar spin state rapidly reaches the steady state value from (4.12). That *s*′_*m*_ would, in the absence of permanent figure torques, rapidly reach this value can be seen from equations (4.8) and (4.11), where for an initial *s*′ _*m*_~ *s*′, the rate of change of the lunar spin is larger than the rate of change of the Earth’s spin by a factor of *A*/*κ*, which is ≥ 10^3^ for *A* ≥ 1.

### 5.1. AM Loss

To estimate the rate at which the evection resonance could drain AM from the Earth-Moon, equation (2.7) is differentiated with respect to time,

(5.1) $${\dot{L}}^{\prime }={\dot{s}}^{\prime }+\kappa {{\dot{s}}^{\prime }}_{m}+{{\dot{L}}^{\prime }}_{\text{orb}}={\dot{s}}^{\prime }+\kappa {{\dot{s}}^{\prime }}_{m}+(\gamma /2){a^{\prime }}^{1/2}{\left(1-\varepsilon_{s}\right)}^{1/2}\left(\frac{{\dot{a}}^{\prime }}{a^{\prime }}-\frac{{\dot{\varepsilon }}_{s}}{1-\varepsilon_{s}}\right).$$

Note that the R.H.S. applies only once *ε*_*s*_ ≥ 0 (post-resonance capture), because for *ε*_*s*_ < 0 (pre-capture), the stationary eccentricity is undefined. Spin rates and the Moon’s semimajor axis are affected only by tides, and since tides alone would conserve system AM, it follows that

(5.2) $${\dot{s}}^{\prime }+\kappa {\dot{s}}_{m}^{\prime }=-(\gamma /2){a^{\prime }}^{1/2}{\left(1-\varepsilon_{s}\right)}^{1/2}\left(\frac{{\dot{a}}^{\prime }}{a^{\prime }}-\frac{{\dot{\varepsilon }}_{T}}{1-\varepsilon_{s}}\right)=-{{\dot{L}}^{\prime }}_{\text{orb},T}.$$

Substituting into (5.1), we confirm that

(5.3) $${\dot{L}}^{\prime }={\dot{L}}^{\prime }{\;}_{\text{orb}}-{\dot{L}}^{\prime }{\;}_{\text{orb},T}=(\gamma /2){a^{\prime }}^{1/2}\left({\dot{\varepsilon }}_{T}-{\dot{\varepsilon }}_{\text{s}}\right)/{\left(1-\varepsilon_{\text{s}}\right)}^{1/2},$$

which again applies only once *ε*_*s*_ ≥ 0. Thus, the change in AM reflects the difference between the rate at which tides change the lunar orbit eccentricity and the rate of change of eccentricity imposed by evection. The rate due to tides, ${\dot{\varepsilon }}_{T}={\dot{\varepsilon }}_{⊕}+{\dot{\varepsilon }}_{m}$, is given by equations (4.6b) and (4.9b), whereas ${\dot{\varepsilon }}_{s}$ can be found by differentiating equation (2.16),

(5.4) $${\dot{\varepsilon }}_{s}\approx \eta \left(\frac{7{\dot{a}}^{\prime }}{4a^{\prime }}-\frac{{\dot{s}}^{\prime }}{s^{\prime }}\right)+6\alpha \frac{{\dot{a}}^{\prime }}{a^{\prime }}.$$

The latter rate is determined primarily by the tidal changes of *a*′ and *s*′, instead of ${\dot{\varepsilon }}_{T}$. The above utilizes the expansion to $O\left(\varepsilon^{2}\right)$ from section 2.4; however, including higher-order terms does not substantially alter the overall behavior so long as α is small.

### 5.2. Tidal Evolution in Resonance With no Libration

**FIGURE 4.**
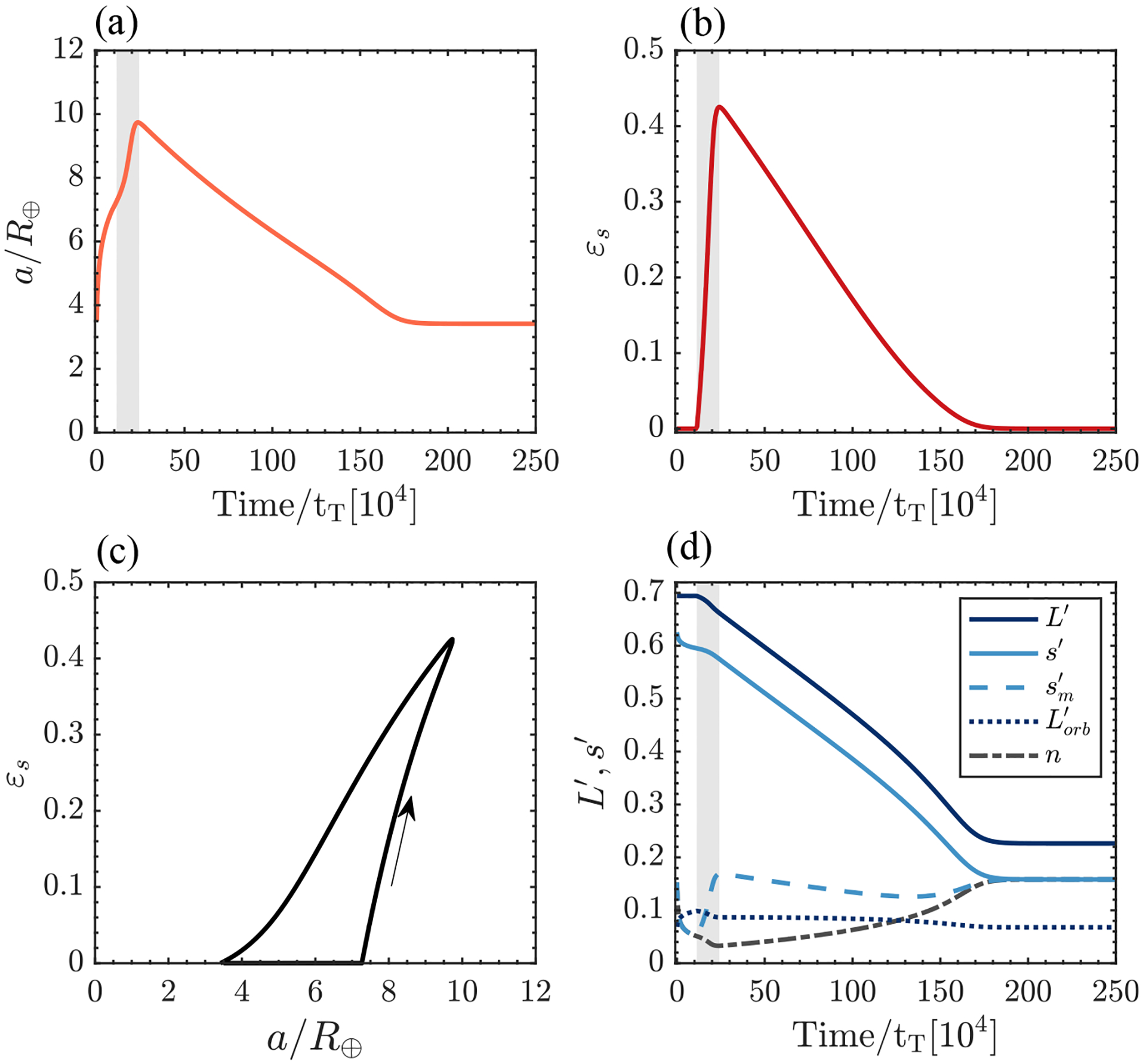
Tidal evolution of the Earth-Moon system in evection with damped libration for *A* = 10 and starting angular momentum *L*_*o*_ = 2*L*_EM_. (a) Scaled lunar semi-major axis, *a*′, as a function of time. (b) The stationary state eccentricity squared, $\varepsilon_{s}\equiv e_{s}^{2}$, vs. time. (c) The stationary state eccentricity squared vs. lunar semimajor axis during the evolution. (d) Time variation of the system angular momentum, *L*, the Earth and Moon spin rates, *s*, *s*_*m*_, the angular momentum of the lunar orbit, *L*_*orb*_ and its mean motion, *n.* The gray region represents the stage where eccentricity is increasing.

Figure 4 illustrates a zero-libration evolution for an initial AM *L*_*o*_ = 2*L*_EM_ and *A* = 10. Additional evolutions for varied *L*_*o*_ and *A* values are presented in Appendix E in the supporting information (Figures SA1 and SA2). Before resonance capture, the semimajor axis (Figure 4a) grows at a rate that decreases with distance, while the eccentricity (Figure 4b) remains zero and the total AM constant (Figure 4d). Once the Moon’s orbit is captured in evection (*ε*_*s*_ ≥ 0), its eccentricity rises (gray regions in Figures 4a, 4b, and 4d), decreasing *L*′_*orb*_ somewhat even though the outward migration temporarily speeds back up. The eccentricity eventually reaches a critical value, *ε*_*c*_, at which outward orbit migration stalls and the orbit begins to contract due to the effect of satellites tides. Soon after, the eccentricity begins to decline as well (the times at which $\dot{a}$ and *ė* vanish are slightly different), and the Moon enters a prolonged contraction phase. If the resonance is maintained with zero libration throughout the evolution, the system would ultimately reach a co-synchronous end-state, *s* = *s*_*m*_ = *n*, with zero eccentricity. However, libration amplitude growth and escape from resonance is predicted long before that state is achieved (see section 6).

**FIGURE 5.**
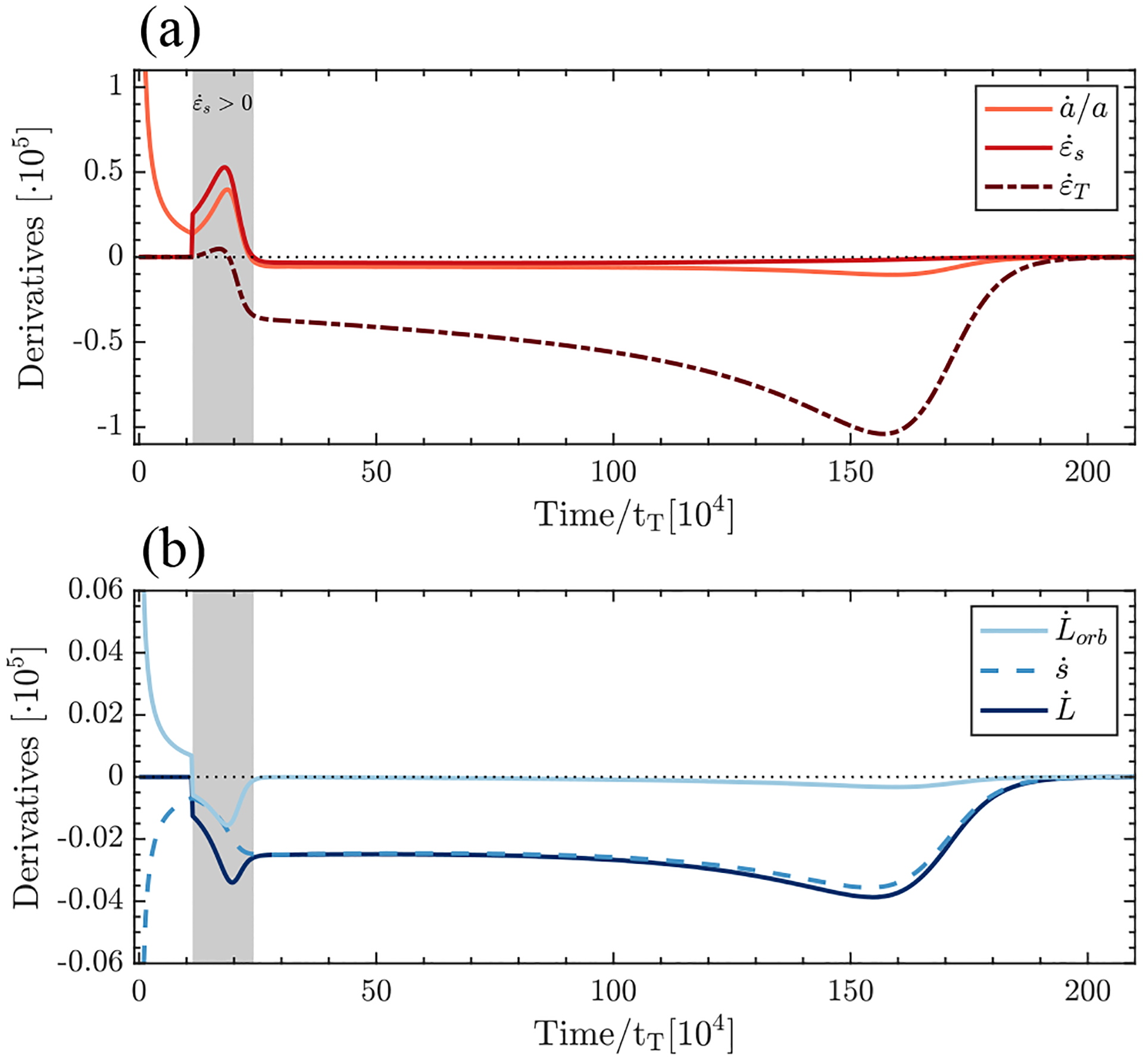
(a) Time variations of the semimajor-axis derivative, ${\dot{a}}^{\prime }/a^{\prime }$, stable eccentricity, ${\dot{\varepsilon }}_{s}$, and eccentricity derivatives due to lunar and earth tides, ${\dot{\varepsilon }}_{T}$; the gray area represents the stage where eccentricity is increasing, ${\dot{\varepsilon }}_{s}>0$.(b) Time variations of the orbital AM, ${{\dot{L}}^{\prime }}_{\text{orb}}$, Earth’s spin, ${\dot{s}}^{\prime }$ and total AM, ${\dot{L}}^{\prime }$, for the evolution in Figure 4. Before resonance capture, the increase in orbital AM is compensated by the decrease in the planet’s spin; hence, the total AM is constant $\left({\dot{L}}^{\prime }=0\right)$ During the outward phase after the resonance capture, the total AM decreases as both the orbital AM and spin rate decrease. During the inward migration stage, ${\dot{\varepsilon }}_{s}<0$, the orbital AM remains relatively constant, while the total AM decreases due to the slowdown of Earth’s spin, ${\dot{L}}^{\prime }\sim \dot{s^{\prime }}$.

The rates ${\dot{a}}^{\prime }/a$, ${\dot{\varepsilon }}_{T}$ and ${\dot{\varepsilon }}_{s}$ during the evolution in Figure 4 are shown in Figure 5a, while Figure 5b displays ${\dot{s}}^{\prime }$, ${\dot{L}}_{\text{orb}}^{\prime }$ and ${\dot{L}}^{\prime }$. The slowdown of the Earth’s spin continues throughout the evolution. The maximum decay rate of *L*′_*orb*_ occurs near the start of lunar contraction, but quickly diminishes to a small value. Throughout the rest of the evolution, the orbital AM remains relatively constant in spite of continued changes in *a*′and *ε*_*s*_. As a result, ${\dot{L}}^{\prime }$ and ${\dot{s}}^{\prime }$ become nearly equal (equation 5.1), i.e., the AM drained from the system by evection is nearly completely reflected in the concomitant slowdown in the Earth’s spin. We now examine each evolutionary stage in greater detail.

#### 5.2.1. Outward Migration

For a Moon in an initially circular orbit outside the Earth’s co-rotation radius ${\dot{s}}^{\prime }$ is negative, while the lunar orbital AM, $L_{\text{orb}}^{\prime }$, increases to compensate so that *dL*′/*dτ* = 0. After resonance capture the Moon’s orbit continues to expand due to tides $\left({\dot{a}}^{\prime }>0\right)$, while evection increases the Moon’s eccentricity (${\dot{\varepsilon }}_{s}>0$; gray area Figure 4) per equation (5.4). Concentrating on just the rate of change of the orbital AM, ${\dot{L}}^{\prime }{\;}_{\text{orb}}$, given by the last term of (5.1) once *ε*_*s*_ > 0, we can write

(5.5) $${\dot{L}}^{\prime }{\;}_{\text{orb}}=\left(L^{\prime }{\;}_{\text{orb}}/2\right)\left[{\dot{a}}^{\prime }/a^{\prime }-{\dot{\varepsilon }}_{s}/\left(1-\varepsilon_{s}\right)\right]\approx \left(L^{\prime }{\;}_{\text{orb}}/2\right)\left[-3{\dot{a}}^{\prime }/4a^{\prime }+{\dot{s}}^{\prime }/s^{\prime }\right]$$

Both terms in the final bracket are negative during this phase, and the AM of the Moon’s orbit decreases with time (Figure 4d) even though its semimajor is increasing.

As evection increases the Moon’s eccentricity, it eventually reaches a critical value, *ε*_*c*_, at which there is a balance between the rates at which Earth and lunar tides alter the Moon’s semimajor axis $({\dot{a}}_{⊕}^{\prime }\approx -{\dot{a}}_{m}^{\prime })$, and the Moon’s orbital expansion stalls at $a^{\prime }=a_{c}^{\prime }$. If the Moon had a very small eccentricity when first captured into resonance at $a_{\text{res}}^{\prime }$, the change in its orbital AM during its migration from there to $a_{c}^{\prime }$ would be $\Delta L_{\text{orb},\text{evec}}^{\prime }=\gamma \left[{a^{\prime }}_{c}^{1/2}{\left(1-\varepsilon_{c}\right)}^{1/2}-{a^{\prime }}_{res}^{1/2}\right]$. For very small initial eccentricity, *η* = Λ*s*′*a*′^7/4^ ≈ 1 and the Earth’s spin upon capture is ${a^{\prime }}_{res}^{7/5}/\Lambda$; while at $a^{\prime }=a_{c}^{\prime }$, $\eta =\Lambda s_{c}^{\prime }/{a^{\prime }}_{c}^{7/4}\approx 1-\varepsilon_{c}$ (neglecting small terms proportional to *α*_0_) so that the corresponding change in the Earth’s spin AM is $\Delta s_{\text{evec}}^{\prime }\approx \left[{a^{\prime }}_{c}^{7/4}\left(1-\varepsilon_{c}\right)-{a^{\prime }}_{res}^{7/4}\right]/\Lambda$. By comparison, the changes in the absence of evection would be simply $\Delta L_{\text{orb},T}^{\prime }=-\Delta s_{T}^{\prime }=\gamma \left({a^{\prime }}_{c}^{1/2}-{a^{\prime }}_{res}^{1/2}\right)$. For the *A* = 10, *L*_*o*_ = 2*L*_EM_ case shown in Figure 4, $a_{\text{res}}^{\prime }\approx 7.30,a_{c}^{\prime }\approx 9.8,\varepsilon_{c}\approx 0.43$ and we find, $\Delta L_{\text{orb},\text{evec}}^{\prime }\approx -0.012$, and $\Delta s_{\text{evec}}^{\prime }\approx -0.027$ for a total loss of $\Delta L_{\text{evec}}^{\prime }\approx -0.039$. Compared to the initial AM of the Earth-Moon system in this case $\left(L_{0}^{\prime }=0.69\right)$, this is only a modest, ~6% reduction. In general, if evection is active only during the Moon’s outbound phase, as was found by Touma and Wisdom (1998), the resulting AM change is small, consistent with prior assumptions of canonical giant impact models (e.g., Canup, 2008).

#### 5.2.2. Inward Migration

Subsequent to stalling, the lunar orbit begins to contract. Provided the resonance condition is maintained and evection continues to control the Moon’s eccentricity (as assumed in the zero libration evolution here), *ε* soon begins to decrease as well. Earth tides further drain *s* as long as, ${\left(1-\varepsilon_{s}\right)}^{-1}{\dot{\varepsilon }}_{s}<{\dot{a}}^{\prime }{\;}_{⊕}/a^{\prime }$ (equation 4.1). It is during this secondary contraction phase that substantial AM may be lost, as was seen in the simulations of CS12.

The contraction of the lunar orbit occurs relatively slowly because the magnitude ${\dot{a}}_{m}^{\prime }<0$ of due to lunar tides is only somewhat larger than the opposing action ${\dot{a}}^{\prime }{\;}_{⊕}$ due to Earth tides. The result is a prolonged period during which AM that is removed from the Earth’s spin by Earth tides can be transferred by the resonance to the Earth’s orbit, with $\dot{s^{\prime }}\sim T^{\prime }$ (as seen in Figure 5b where $\dot{s^{\prime }}\sim \dot{L^{\prime }}$). The changes in the components of the Earth-Moon AM during this phase would be ${\Delta L^{\prime }{\;}_{\text{orb}}|}_{\text{evec}}=\gamma \left[{a^{\prime }}_{\text{esc}}^{1/2}{\left(1-\varepsilon_{\text{esc}}\right)}^{1/2}-{a^{\prime }}_{c}^{1/2}{\left(1-\varepsilon_{c}\right)}^{1/2}\right]$ and $\Delta s^{\prime }{\;}_{\text{evec}}=\left[{a^{\prime }}_{esc}^{7/4}\left(1-\varepsilon_{esc}\right)-{a^{\prime }}_{c}^{7/4}\left(1-\varepsilon_{c}\right)\right]/\Lambda$, where *a*′_*esc*_, *ε*_*esc*_ are the semimajor axis and eccentricity (squared) at the time of resonance escape. For the particular evolution shown in Figure 4 (with *A* = 10, *L*_0_ = 2*L*_EM_), the system AM decreases to that of the current Earth-Moon system ${L^{\prime }}_{\text{EM}}=0.35$ at Time/*t*_*T*_ = 1.4 × 10^6^. For resonance escape to occur at this point implies $a_{\text{esc}}^{\prime }=4.7$ and *ε*_*esc*_ = 0.06.

From Figure 4d (and also Figures SA1–SA3 in Appendix E in the supporting information), it can be seen that the longer the Moon remains in resonance, the greater the reduction in *L*, so that the final AM achieved via formal evection will be set by the timing of resonance escape. Escape can occur if the adiabatic condition is violated (so that the timescale for tidally driven changes in *ε* becomes short compared to the resonant libration timescale), or if the libration amplitude grows and exceeds the maximum amplitude of *π*/2 consistent with resonant libration. If escape never occurred, evection would drain the system’s AM until the dual synchronous state is achieved. The limiting final AM in this case is found by setting *s*′, $s_{m}^{\prime }=s_{\text{sync}}^{\prime }={a^{\prime }}^{-3/2}$ and *ε*_*s*_ = 0, viz.,

(5.6) $$L_{\text{sync}}^{\prime }=(1+\kappa )/{a^{\prime }}_{\text{sync}}^{3/2}+\gamma {a^{\prime }}_{\text{sync}}^{1/2},$$

where $a_{\text{sync}}^{\prime }$ is the final semimajor axis. This will be right at the inner boundary of the resonance where *ε*_*s*_ can go to zero and is obtained by setting $\Lambda s_{\text{sync}}^{\prime }/{a^{\prime }}_{\text{sync }}^{7/4}=\eta_{\text{sync}}=1+4\alpha_{\text{sync}}$, and then solving for $a_{\text{sync}}^{\prime }≅\Lambda^{4/13}=3.416$. Equation (5.6) then gives $L_{\text{sync}}^{\prime }=0.226$, which is substantially less than that of the current Earth-Moon $\left(L_{\text{EM}}^{\prime }=0.35\right)$. In addition to being inconsistent with the Earth-Moon AM, a dual synchronous state would also be unstable, because further slowing of the Earth’s spin by direct solar tides would eventually cause synchronous orbit to drift beyond the Moon, which would then tidally evolve inward. Clearly, this full evolution in evection never occurred for the Earth-Moon pair, and indeed in section 6, we predict much earlier resonance escape.

### 5.3. Tidal Stationary States

The above baseline evolution adopts the stationary state eccentricity in the absence of tides. If tides are included as in equations (4.4a,b), equation (4.14) describing libration about the stationary state is replaced by

(5.7) $$\ddot{\theta }=-40\chi^{2}\alpha \left(\varepsilon_{*}+5\alpha \text{cos}2\theta \right)\text{sin}2\theta +2\chi \left({\dot{\varepsilon }}_{T}-{\dot{\varepsilon }}_{*}-5\dot{\alpha }\text{cos}2\theta \right)$$

and the angle, *θ*_*s*_, for which $\ddot{\theta }$ vanishes satisfies,

(5.8) $$20\chi \alpha \left(\varepsilon_{*}+5\alpha \text{cos}2\theta_{s}\right)\text{sin}2\theta_{s}={\dot{\varepsilon }}_{T}-{\dot{\varepsilon }}_{*}-5\dot{\alpha }\text{cos}2\theta_{s}.$$

The angle *θ*_*s*_ represents an offset from the *y*-axis of the stationary state around which stable libration occurs that is due to the effects of tides. If the tidal rates are small enough that the offset angle is small and cos2*θ*_*s*_ ~ 1, $\text{sin}2\theta_{s}\approx \left({\dot{\varepsilon }}_{T}-{\dot{\varepsilon }}_{*}-5\dot{\alpha }\right)/20\chi \alpha \left(\varepsilon_{*}+5\alpha \right)$; if instead tidal rates are fast and the offset is large so that cos2*θ*_*s*_ is small, $\text{sin}2\theta_{s}\approx \left({\dot{\varepsilon }}_{T}-{\dot{\varepsilon }}_{*}\right)/20\chi \alpha \varepsilon_{*}$. However, since the maximum value of |sin2*θ*_*s*_| is unity when *θ*_*s*_ = ± 45°, there can be no stationary angle (and thus no stable libration) if $\left|{\dot{\varepsilon }}_{T}-{\dot{\varepsilon }}_{*}\right|>20\chi \alpha \varepsilon_{*}$.

Using the low cos2*θ*_*s*_ approximation and neglecting terms proportional to *α* and $\dot{a}$, equation (4.4a) becomes $\dot{\varepsilon }\approx -\left({\dot{\varepsilon }}_{T}-{\dot{\varepsilon }}_{*}\right)\varepsilon /\varepsilon_{*}+{\dot{\varepsilon }}_{T}$, and differentiating yields

(5.9a) $$\ddot{\varepsilon }\approx [-\dot{\varepsilon }+(\dot{\varepsilon }*/\varepsilon *)\varepsilon ]\left({\dot{\varepsilon }}_{T}-\dot{\varepsilon }*\right)/\varepsilon *+(1-\varepsilon /\varepsilon *){\ddot{\varepsilon }}_{T}+(\varepsilon /\varepsilon *)\ddot{\varepsilon }*.$$

Ignoring ${\ddot{\varepsilon }}_{T}$, ${\ddot{\varepsilon }}_{*}$, using the $\dot{\varepsilon }$ expression above equation (5.9a) and requiring $\ddot{\varepsilon }\to 0$ to suppress oscillations, results in

(5.9b) $$\left(\varepsilon /\varepsilon_{*}-1\right)\left({\dot{\varepsilon }}_{T}-{\dot{\varepsilon }}_{*}\right){\dot{\varepsilon }}_{T}/\varepsilon_{*}\approx 0.$$

Assuming nonzero tidal rates $\left({\dot{\varepsilon }}_{T}\neq 0\right)$, satisfying this condition implies *ε*_*s*_ ≈ ε_*_, vs. *ε*_*s*_ ≈ ε_*_+5*α* found in the $O\left(\varepsilon^{2}\right)$ expansion in section 2.4. Thus, the stationary eccentricity is relatively unaffected by an increasing stationary offset angle imposed by tides. Note that substituting these state parameters into equations (4.4a,b) will not give zero values for ${\dot{E}}_{S}$ and ${\dot{\theta }}_{S}$ because they are now slowly changing quasi-steady states.

## 6. Evolution: Finite Libration and Resonance Escape

Until now, the orbit evolution has been artificially constrained to zero libration. On the other hand, Touma and Wisdom (1998) found that the Moon escapes evection soon after it reaches the distance where $\dot{a}=0$. At this point, they found that the resonance libration amplitude begins to rapidly increase until the system leaves resonance. Escapes were also reported by CS12, although much later in the evolution during the orbital contraction phase. In this section, we explore how tides affect the libration behavior, libration amplitude growth, and the expected timing of resonant escape.

At the turn-around point of a level curve, $\partial \tilde{J}/{\partial \varepsilon |}_{\Theta }=0$. From equation (2.20a), this implies that

(6.1) $$\varepsilon_{\Theta }=\varepsilon_{*}+5\alpha \text{cos}2\Theta$$

where *ε*_Θ_ denotes the eccentricity at turn-around, and we have assumed $\dot{a}$ is small during a libration cycle. Substituting into $\tilde{J}$ then leads to

(6.2a,b) $$\varepsilon_{\Theta }^{2}=-2\tilde{J};\text{cos}2\Theta =\left(\sqrt{-2\tilde{J}}-\varepsilon_{*}\right)/5\alpha .$$

### 6.1. Libration Amplitude Variation

To examine the behavior of the libration amplitude on an oscillation timescale, we wish to integrate equation (5.7) including eccentricity variations over a libration cycle. We consider a case where the libration amplitude is small and retain only terms linear in *θ* to find,

(6.3) $$\ddot{\theta }+\omega^{2}\theta \approx 2\chi \left({\dot{\varepsilon }}_{T}-{\dot{\varepsilon }}_{s}\right)\equiv F,$$

where again *ω*^2^ ≡ 80*χ*^2^*αε*_*s*_. As in equation (4.14), this resembles a harmonic oscillator of frequency *ω*, but now with an additional forcing term, F, due to tides. The solution to equation (6.3) has two parts: a homogeneous solution, *θ*_*h*_ = Θsin*ωτ*, equal to that of the unforced equation, and a particular solution,

(6.4) $$\theta_{p}=-\frac{\text{cos}\omega \tau }{\omega }\int F(\tau )\text{sin}\;\omega \tau d\;\tau +\frac{\text{sin}\omega \tau }{\omega }\int F(\tau )\text{cos}\;\omega \tau d\;\tau .$$

The F(τ) term has an oscillating part through its *ε* dependence over a libration cycle and can be expanded to lowest order around its value $F_{s}\equiv F\left(a^{\prime },s^{\prime },\varepsilon_{s}\right)$ at *ε*_*s*_, i.e.,

(6.5a) $${\dot{\varepsilon }}_{T}\approx {\dot{\varepsilon }}_{T}\left(\varepsilon_{s}\right)+\frac{\partial {\dot{\varepsilon }}_{T}}{\partial \varepsilon }\left(\varepsilon -\varepsilon_{s}\right)+\cdots ;{\dot{\varepsilon }}_{s}\approx {\dot{\varepsilon }}_{s}\left(\varepsilon_{s}\right)+\frac{\partial {\dot{\varepsilon }}_{s}}{\partial \varepsilon }\left(\varepsilon -\varepsilon_{s}\right)+\cdots ,$$

implying

(6.5b) $$F\left(a^{\prime },s^{\prime },\varepsilon \right)\approx F_{s}+{\frac{\partial F}{\partial \varepsilon }|}_{\varepsilon_{s}}\left(\varepsilon -\varepsilon_{s}\right)+\cdots .$$

The lead term results in a particular solution, $\theta_{s}=F_{s}/\omega^{2}$ that reduces to the tidal stationary angle of section 5.3. However, the second term produces a time-varying particular solution describing libration.

The linearized version of equation (2.21a) for small *θ* and *ε* ≈ *ε*_*s*_ reads $\dot{\varepsilon }\approx -40\chi \alpha \varepsilon_{s}\theta$, and utilizing the homogeneous solution for *θ* integrates to

(6.6) $$\varepsilon -\varepsilon_{s}\approx 40\alpha \varepsilon_{s}(\chi /\omega )\Theta_{o}\text{cos}\omega \tau ,$$

where Θ_*o*_ represents the libration amplitude at the start of a given cycle when *ωτ* = − *π*/2. Substituting equations (6.5b) and (6.6) into equation (6.4) and integrating, we get the time-varying particular solution,

(6.7) $$\theta_{p}=20\alpha \varepsilon_{s}\frac{\chi \partial F}{\omega^{2}\partial \varepsilon }\Theta_{o}\tau \text{sin}\omega \tau .$$

Combining and arranging terms gives the variation of *θ* with respect to the tidal stationary offset angle *θ*_*s*_,

(6.8) $$\theta -\theta_{s}=\theta_{h}+\theta_{p}\approx \Theta_{0}\left[1+20\alpha \varepsilon_{s}\frac{\chi \;\partial F}{\omega^{2}\;\partial \varepsilon }\tau \right]\text{sin}\omega \tau ,$$

and it is seen that libration relative to the offset angle will change with time due to the ∂F/∂ε term, i.e., due to the variation in $\left({\dot{\varepsilon }}_{T}-{\dot{\varepsilon }}_{s}\right)$ during a libration cycle due to small changes in *ε*. There is a resulting change, $\Delta \Theta =40\pi \alpha \varepsilon_{s}\left(\chi /\omega^{3}\right)(\partial F/\partial \varepsilon )\Theta_{0}$, in the oscillation amplitude after a complete cycle, Δ*τ* = 2*π*/*ω*. This updated value then applies to the next cycle, etc., implying,

(6.9) $$\frac{1\;d\Theta }{\Theta \;d\tau }=20\alpha \varepsilon_{s}\frac{\chi \;\partial F}{\omega^{2}\;\partial \varepsilon }=40\alpha \varepsilon_{s}{\left(\frac{\chi }{\omega }\right)}^{2}\left(\frac{\partial {\dot{\varepsilon }}_{T}}{\partial \varepsilon }-\frac{\partial {\dot{\varepsilon }}_{s}}{\partial \varepsilon }\right)=\frac{1}{2}\left(\frac{\partial {\dot{\varepsilon }}_{T}}{\partial \varepsilon }-\frac{\partial {\dot{\varepsilon }}_{s}}{\partial \varepsilon }\right),$$

where the last step uses the *ω* definition.

**FIGURE 6.**
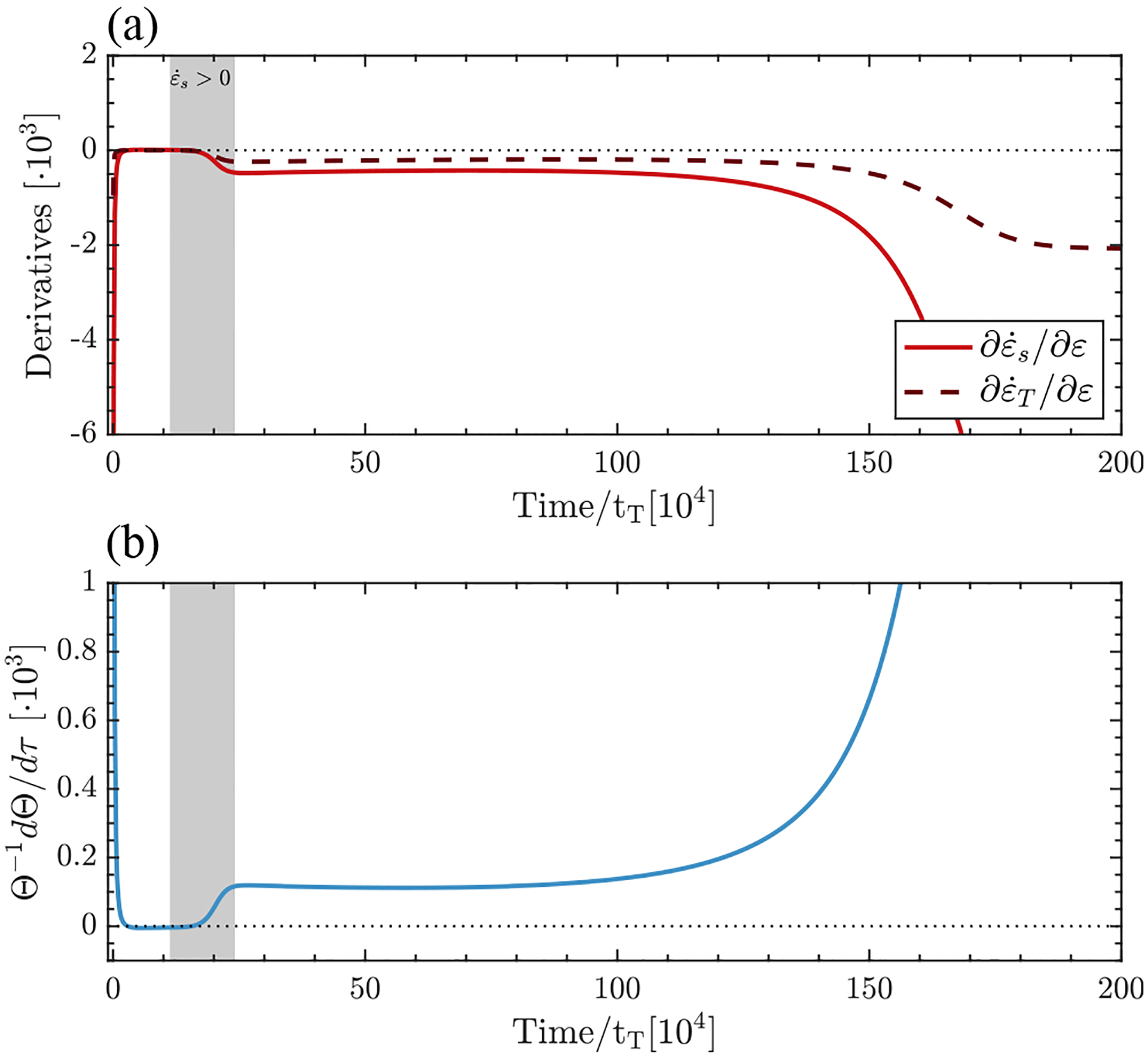
(a) Partial derivatives of $\partial {\dot{\varepsilon }}_{\text{s}}/\partial \varepsilon$ (solid red) and $\partial {\dot{\varepsilon }}_{T}/\partial \varepsilon$ (dashed dark red) of the evolution depicted in Figure 4. (b) Rate of change for the libration amplitude, $\dot{\Theta }/\Theta$. during most of the outward migration (gray area) the libration amplitude decreases, maintaining formal resonance. Near the turnaround point, the libration amplitude increases, promoting resonance escape.

Thus, whether the libration amplitude grows or damps depends on the sign of $\left(\partial {\dot{\varepsilon }}_{T}/\partial \varepsilon -\partial {\dot{\varepsilon }}_{s}/\partial \varepsilon \right)$. For example, if both $\partial {\dot{\varepsilon }}_{T}/\partial \varepsilon$ and $\partial {\dot{\varepsilon }}_{s}/\partial \varepsilon$ are positive (as occurs during the initial phase of expansion in resonance, see Figure 6a), then the libration amplitude will damp if the rate of change in the Moon’s eccentricity due to tides increases more slowly with *e* (i.e., *ε*) than does the rate of change of the stationary eccentricity. Conversely, once $\partial {\dot{\varepsilon }}_{T}/\partial \varepsilon >\partial {\dot{\varepsilon }}_{s}/\partial \varepsilon$ (which occurs near the stagnation point, $\dot{a}=0$), the libration amplitude increases with time (e.g., Figure 6a).

The partial derivatives depend on the specific tidal model employed. For the model of Mignard (1980) used here

(6.10) $${a^{\prime }}^{8}\frac{\partial {\dot{\varepsilon }}_{T}}{\partial \varepsilon }=\left(s^{\prime }+As_{m}^{\prime }\right){a^{\prime }}^{3/2}\left(1+\frac{\varepsilon }{{\tilde{g}}_{1}}\frac{\partial {\tilde{g}}_{1}}{\partial \varepsilon }+\frac{5\varepsilon }{1-\varepsilon }\right)g_{1}-(1+A)\left(1+\frac{\varepsilon }{{\tilde{g}}_{2}}\frac{\partial {\tilde{g}}_{2}}{\partial \varepsilon }+\frac{13\varepsilon /2}{1-\varepsilon }\right)g_{2},$$

(6.11) $$\frac{\partial {\dot{\varepsilon }}_{s}}{\partial \varepsilon }\approx \left(\frac{7\eta }{4}+6\alpha \right)\frac{\partial }{\partial \varepsilon }\left(\frac{{\dot{a}}^{\prime }}{a^{\prime }}\right)-\frac{\eta \partial \dot{s^{\prime }}}{s^{\prime }\partial \varepsilon },$$

while taking the derivatives of $\dot{a^{\prime }}/a^{\prime }$ and $\dot{s^{\prime }}$ gives

(6.12) $${a^{\prime }}^{8}\frac{\partial }{\partial \varepsilon }\left(\frac{{\dot{a}}^{\prime }}{a^{\prime }}\right)=\left(s^{\prime }+As_{m}^{\prime }\right){a^{\prime }}^{3/2}\left(\frac{1}{{\tilde{f}}_{1}}\frac{\partial {\tilde{f}}_{1}}{\partial \varepsilon }+\frac{6}{1-\varepsilon }\right)f_{1}-(1+A)\left(\frac{1}{{\tilde{f}}_{2}}\frac{\partial {\tilde{f}}_{2}}{\partial \varepsilon }+\frac{15/2}{1-\varepsilon }\right)f_{2},$$

(6.13) $$\frac{\partial \dot{s^{\prime }}}{\partial \varepsilon }=-\frac{1}{2}\gamma a^{\prime 1/2}\frac{\partial }{\partial \varepsilon }\left({\left(1-\varepsilon \right)}^{1/2}\frac{{\dot{a}}_{⊕}^{\prime }}{a^{\prime }}-\frac{1}{{\left(1-\varepsilon \right)}^{1/2}}{\dot{\varepsilon }}_{⊕}\right)=\frac{1}{2}\gamma a^{\prime 1/2}(\frac{1}{2{\left(1-\varepsilon \right)}^{1/2}}\left(\frac{{\dot{a}}_{⊕}^{\prime }}{a^{\prime }}+\frac{{\dot{\varepsilon }}_{⊕}}{1-\varepsilon }\right)-{\left(1-\varepsilon \right)}^{1/2}\left(\frac{\partial }{\partial \varepsilon }\frac{{\dot{a}}_{⊕}^{\prime }}{a^{\prime }}-\frac{1}{1-\varepsilon }\frac{\partial {\dot{\varepsilon }}_{⊕}}{\partial \varepsilon }\right)),$$

where the *f* and *g* polynomials and their derivatives (Table 2) are to be evaluated for *ε* = *ε*_*s*_. Setting *A* = 0 in equations (6.10) and (6.12) provides the Earth-only tidal expressions needed for $\partial {\dot{s}}^{\prime }/\partial \varepsilon$. Analogous expressions for the case of synchronous lunar rotation maintained by a permanent figure torque are provided in Appendix D in the supporting information.

**FIGURE 7.**
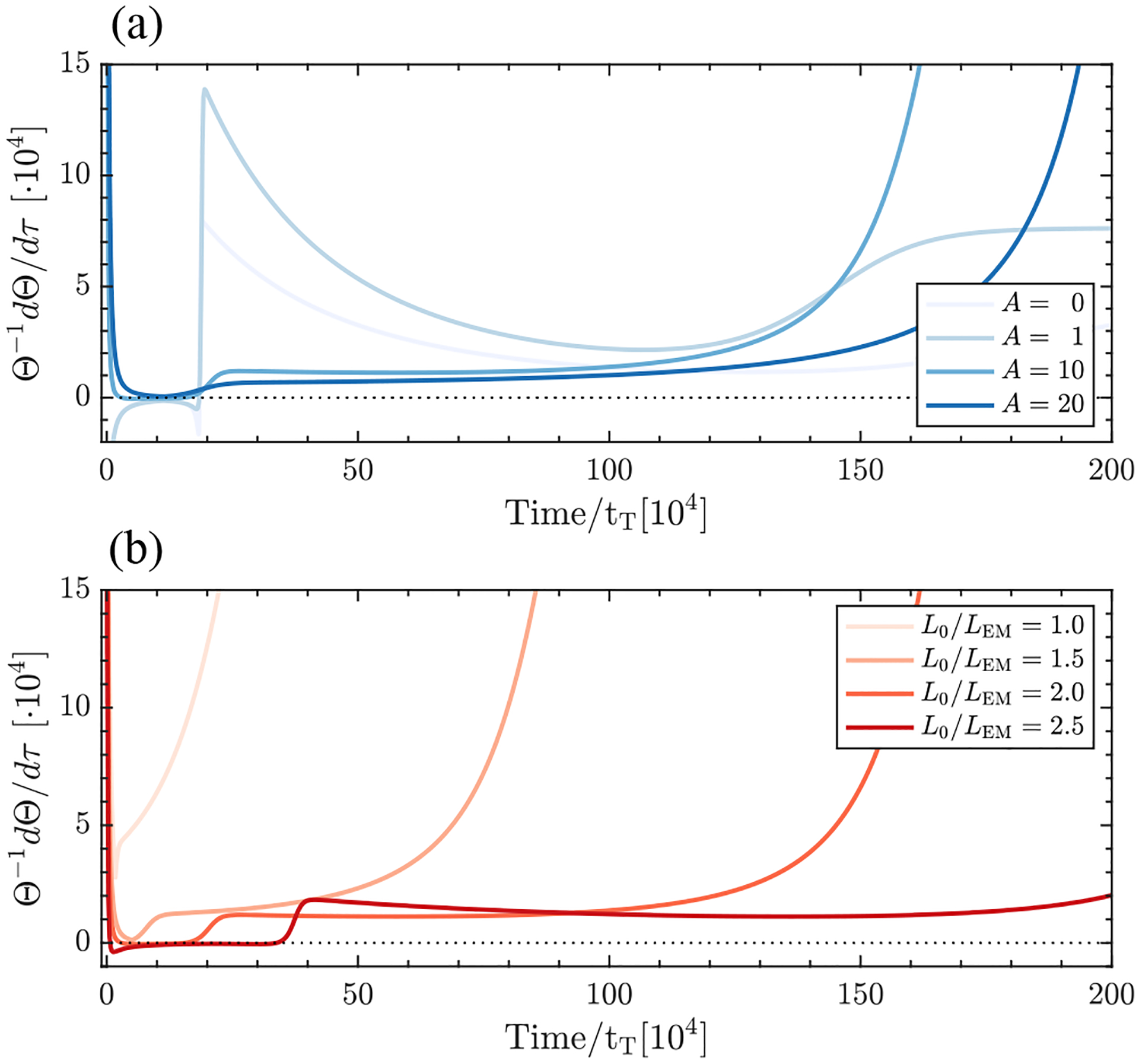
The evolution of the rate of change of libration amplitude $\left(\dot{\Theta }/\Theta \right)$ for (a) varied *A* values for *L*_0_ = 2*L*_EM_ and (b) With larger A values, the transition between varied *L*_0_/*L*_EM_ values for *A* = 10. With larger *A* values, the transition between libration amplitude damping and excitation is more gradual. With larger initial AM values, the libration amplitude damping stage, which promotes resonance occupancy, is longer.

**FIGURE 8.**
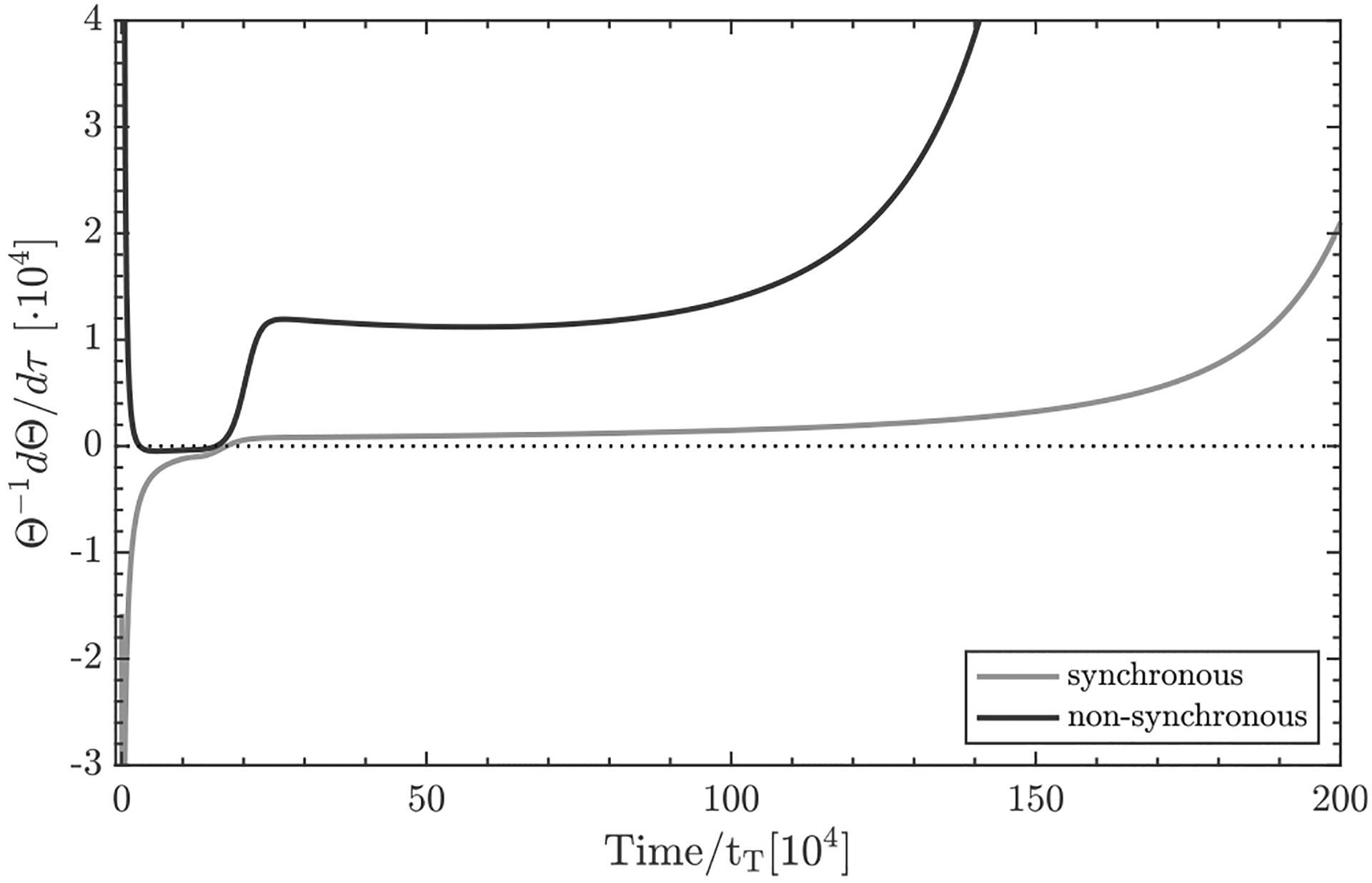
Evolution of the rate of change of libration amplitude $\left(\dot{\Theta }/\Theta \right)$ for synchronous rotation maintained by a permanent figure torque with *L*_*o*_ = 2*L*_EM_ and *A* = 10 (gray line), with non-synchronous rotation case shown for comparison (black line). The libration amplitude excitation for the synchronous rotation case is more gradual compared to the nonsynchronous case; hence, the resonance escape is delayed. Note that the AM removal rate in the synchronous case is lower than the nonsynchronous case (see Figure A3); hence, despite this delay, the overall amount of AM removed by evection is reduced.

Figure 6 displays the partial derivative behaviors and Θ^−1^*d*Θ/*dτ* for the baseline evolution shown in Figure 4 (with *A* = 10, *Lo* = 2*L*_EM_). Figure 7 shows Θ^−1^ *d*Θ/*dτ* for varied *A* values for *L*_*o*_ = 2*L*_EM_, and for varied *L*_*o*_ with *A* = 10, all for a nonsynchronously rotating Moon. Figure 8 contrasts Θ^−1^*d*Θ/*dτ* for synchronous vs. non-synchronous rotation cases, both with *A* = 10 and *L*_*o*_ = 2*L*_EM_. Across all parameter choices, libration amplitude growth (i.e., Θ^−1^*d*Θ/*dτ* > 0) is predicted for Mignard tides during the lunar orbital contraction phase.

**FIGURE 9.**
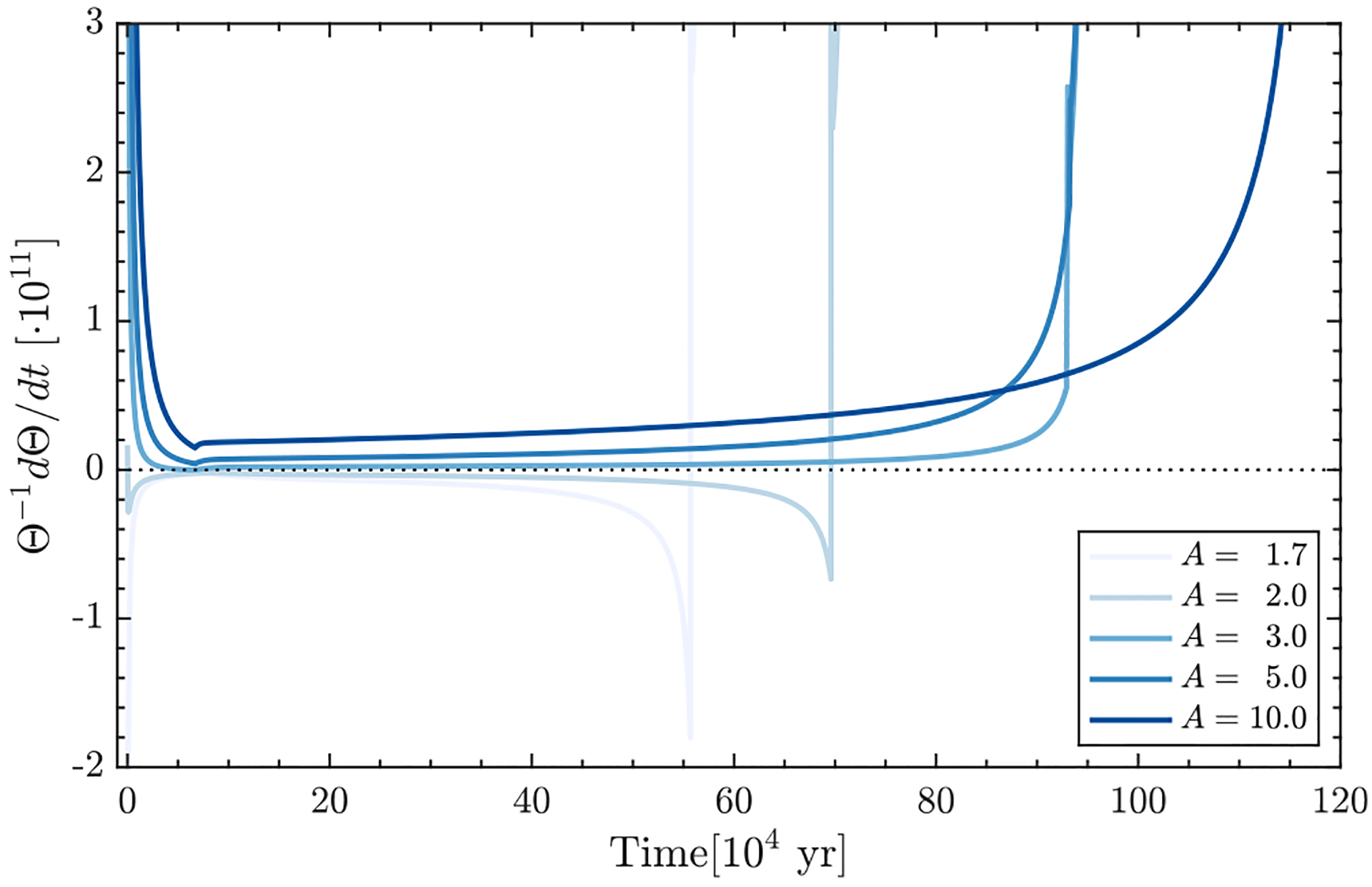
Evolution of the rate of change of libration amplitude (Θ/ Θ) using the constant-*Q* tidal model for a synchronously rotating moon, with tidal expressions and associated tidal *A* constant defined by eqns. (12) and (21) to (40) in Wisdom and Tian (2015), for *L*_*o*_ = 2*L*_EM_, *Q*_⊕_ = 400, and varied *A* values. With a constant-*Q* model and *A* = 1.7 and 2, equation (6.9) predicts an extended period of libration amplitude damping (Θ/ Θ < 0) even as the Moon’s semi-major axis contracts (orbit contraction for these cases commences at *t* ≤ 15 [10^4^ year]). This implies protracted resonance occupancy, consistent with simulations of Wisdom & Tian for this narrow range of *A* values (e.g., their Figures 2 and 9). In contrast, for *A* ≥ 3 (darker blue lines) increasingly strong amplitude excitation is predicted, suggesting limited resonance occupancy. Wisdom & Tian found minimal or no libration in formal evection for constant-*Q* tides and these larger *A* values.

Before estimating when libration amplitude growth would lead to resonance escape with Mignard tides (section 6.2 below), we briefly consider application of equation (6.9) to the constant lag angle/constant-*Q* tidal model utilized in Wisdom and Tian (2015). Figure 9 shows the predicted behavior of Θ^−1^*d*Θ/*dt* for a baseline evolution (i.e., with *ε* = *ε*_*s*_ and *θ* = 0) that adopts the tidal expressions for a synchronously rotating Moon as given in Wisdom and Tian’s equations (21) through (40), with *A* now defined in their eqn. (12). It can be seen that for the *A* = 1.7 and 2.0 cases (light blue curves in Figure 9), equation (6.9) predicts an extended period of libration amplitude damping that persists even as the Moon’s orbit contracts, implying resonance stability. This is consistent with protracted resonance occupancy, decreasing libration amplitude, and large AM modification seen for these *A* values in both the simplified models and full integrations of Wisdom and Tian (e.g., their Figures 2, 3, 5, and 9). However, outside this narrow range of *A*, equation (6.9) predicts libration amplitude excitation even prior to lunar orbit contraction (darker blue curves in Figure 9), suggesting limited resonance stability. For this regime, Wisdom and Tian indeed found minimal or no formal resonance occupancy with constant-*Q* tides.

Predictions from the idealized solutions developed here thus appear qualitatively consistent with results of more complete integrations with regards to formal resonance occupancy (although our methods do not allow us to assess the nonlibrating “limit cycle” behavior seen in Wisdom and Tian, a point we return to in section 7). That constant-*Q* model evolutions find prolonged damped libration in resonance for a narrow range of *A* values (Figure 9; Wisdom & Tian, 2015), while evolutions with Mignard tides do not (Figures 7 and 8 and section 6.2), thus appears to be due to differences in the tidal models themselves rather than to other differences between this work and that of Wisdom and Tian (e.g., different evolution methods, inclusion of finite terrestrial obliquity, and/or lunar inclination in their integrations, etc.). As the Moon’s orbit contracts, $\partial {\dot{\varepsilon }}_{T}/\partial \varepsilon$ and $\partial {\dot{\varepsilon }}_{s}/\partial \varepsilon$ are negative for both the constant-*Q* and Mignard models. However, for constant-*Q* tides with *A* = 1.7 and 2.0, $\left|\partial {\dot{\varepsilon }}_{T}/\partial \varepsilon \right|>\left|\partial {\dot{\varepsilon }}_{s}/\partial \varepsilon \right|$ for an extended period during orbit contraction, implying damping, while for Mignard tides $\left|\partial {\dot{\varepsilon }}_{T}/\partial \varepsilon \right|<\left|\partial {\dot{\varepsilon }}_{s}/\partial \varepsilon \right|$, implying excitation (e.g., Figure 6a). Beyond this narrow range of *A*, both the constant-*Q* and Mignard models have $\left|\partial {\dot{\varepsilon }}_{T}/\partial \varepsilon \right|<\left|\partial {\dot{\varepsilon }}_{s}/\partial \varepsilon \right|$ during contraction, implying libration amplitude excitation. Differences in evolution rates (i.e., *de*/*dt*, *da*/*dt*) between the constant-*Q* and Mignard tidal models are most pronounced for high-eccentricity orbits, and so it is not surprising that the divergent outcomes occur for low *A* cases in which the peak eccentricities are highest. It is also for high-*e* orbits that the assumption of a constant lag-angle (inherent to the constant-*Q* model) is perhaps most suspect.

### 6.2. Excitation and Resonance Escape

We now return to libration excitation and the timing of escape for Mignard tides. Per Figures 6–8, during most of the Moon’s outbound evolution in evection the libration amplitude is damped (i.e., $\dot{\Theta }/\Theta <0$) or undergoes only weak excitation. However, as the Moon approaches the turn-around point in semi-major axis, there is a transition to increasing excitation. For low *A*, damped libration in the outbound phase rapidly transitions to excitation near the stall point (Figures 6 and 7a), reminiscent of the behavior seen in Touma and Wisdom (1998), suggesting that escape from resonance is likely to occur near this point, depending on the initial libration amplitude following capture. For larger values of *A,* libration amplitude growth past the stall point is more modest (Figure 7a); however, $\dot{\Theta }/\Theta$ remains positive throughout the Moon’s subsequent orbital contraction, and its magnitude generally increases with time. This implies that resonant escape will occur well before the dual-synchronous end state is reached in the high-*A* cases as well.

It is instructive to consider how cyclic variations in tidal strength lead to amplitude changes during a single libration cycle. First consider the lead constant term, $F_{s}=2\chi \left[{\dot{\varepsilon }}_{T}\left(\varepsilon_{s}\right)-{\dot{\varepsilon }}_{s}\left(\varepsilon_{s}\right)\right]$, in the forcing function from equation (6.5b). A constant ${\dot{\varepsilon }}_{T}\left(\varepsilon_{s}\right)$ during a state’s counter-clockwise traverse of the upper level curve branch tries to push the trajectory across level curves. For specificity, on a level curve with turn-around points ±Θ_*o*_ during the orbital contraction phase, ${\dot{\varepsilon }}_{T}\left(\varepsilon_{s}\right)<0$, and the trajectory on the upper branch drifts downward toward level curves with more negative $\tilde{J}$ (see Figure 1f). As a result, it encounters a turning point at Θ slightly less than Θ_*o*_. However, over the return, rightward trip on the lower branch, its continued downward motion causes the state to drift across level curves of higher $\tilde{J}$, reversing the process. The net result is a trajectory path that resembles a level curve, but whose point of symmetry is shifted off the *y*-axis to an angle $\sim {\dot{\varepsilon }}_{T}\left(\varepsilon_{s}\right)/40\chi \alpha \varepsilon_{s}<0$ (see equation (5.8) for the case of a small offset angle, and note that we define *θ* as the angle from the *y*-axis in the direction of the negative *x*-axis, so that a negative offset angle lies in quadrant I of our coordinate system). This same path is repeated on future cycles unless there is a change in *ε*_*s*_. A similar situation occurs for a constant ${\dot{\varepsilon }}_{s}\left(\varepsilon_{s}\right)<0$ with ${\dot{\varepsilon }}_{T}=0$, where a level curve with given turn-around points ±Θ_*o*_ migrates down the *y*-axis; in this case, there is a change in the Jacobi value associated with Θ_*o*_ found from equation (6.2b), viz., $\tilde{J}=-{\left(\varepsilon *+5\alpha \text{cos}2\Theta_{o}\right)}^{2}/2$.This causes the state’s position to be crossed by level curves with larger libration amplitudes, Θ > Θ_*o*_ during its upper counter-clockwise traverse, but by curves of Θ < Θ_*o*_ on its rightward lower return. In this case, the trajectory again resembles the shape of a level curve shifted off the *y*-axis by $\sim -{\dot{\varepsilon }}_{s}\left(\varepsilon_{s}\right)/40\chi \alpha \varepsilon_{s}>0$. Since during contraction, the magnitude of ${\dot{\varepsilon }}_{T}$ is generally larger than ${\dot{\varepsilon }}_{s}$ (see Figure 5a), their combined influence yields the negative stationary state angle. But, for ${\dot{\varepsilon }}_{T}$ and ${\dot{\varepsilon }}_{s}$ that are constant during a libration cycle, there is no net change in libration amplitude.

Now consider the second term in equation (6.5b), $\partial F/{\partial \varepsilon |}_{\varepsilon_{\text{s}}}\left(\varepsilon -\varepsilon_{\text{s}}\right)=2\chi \left[\partial {\dot{\varepsilon }}_{T}/\partial \varepsilon \left(\varepsilon_{s}\right)-\partial {\dot{\varepsilon }}_{s}/\partial \varepsilon \left(\varepsilon_{s}\right)\right]\left(\varepsilon -\varepsilon_{s}\right)$, describing cyclic variations of the tidal strengths. Figure 6a displays the partial derivatives $\partial {\dot{\varepsilon }}_{T}/\partial \varepsilon$, $\partial {\dot{\varepsilon }}_{s}/\partial \varepsilon$ during the evolution shown in Figure 4. Although during most of the orbit contraction phase the magnitude of ${\dot{\varepsilon }}_{T}$ exceeds that of ${\dot{\varepsilon }}_{s}$ (see Figure 5a), in Figure 6a, we see that $\left|\partial {\dot{\varepsilon }}_{s}/\partial \varepsilon \right|>\left|\partial {\dot{\varepsilon }}_{T}/\partial \varepsilon \right|$ and that both derivatives are negative. Thus, the quantity $\left[\partial {\dot{\varepsilon }}_{T}/\partial \varepsilon \left(\varepsilon_{s}\right)-\partial {\dot{\varepsilon }}_{s}/\partial \varepsilon \left(\varepsilon_{s}\right)\right]$ is positive during contraction, so that when (*ε* − *ε*_*s*_) > 0, there is positive forcing, while when (*ε* − *ε*_*s*_) < 0, the libration amplitude is damped. However, the two effects do not exactly compensate because (*ε* − *ε*_*s*_) > 0 for proportionally more of the libration cycle, and consequently, a cycle finishes with a larger amplitude then when it started.

In the early outbound phase in evection, libration amplitude is typically damped. If there were no tidal change in *ε*_*s*_ in this phase, equation (6.9) would reduce to $\dot{\Theta }/\Theta \to (1/2)\partial {\dot{\varepsilon }}_{T}/\partial \varepsilon$, and when $\partial {\dot{\varepsilon }}_{T}/\partial \varepsilon <0$ (as implied by damping), the libration amplitude could be driven to a vanishing small quantity. However, if ${\dot{\varepsilon }}_{s}\neq 0$, there is a limit to this. The eccentricity of the upper libration path at the *y*-axis is $\varepsilon_{s}+\sqrt{\varepsilon_{s}^{2}+2\tilde{J}}$, implying a path half-width of $w=\sqrt{\varepsilon_{s}^{2}-\varepsilon_{\Theta }^{2}}$, the final form employing equation (6.2a). Assuming Θ is small, cos2Θ ≈ 1 − 2Θ^2^ and equation (6.1) reads *ε*_Θ_ ≈ *ε*_*s*_ − 10*α*Θ^2^. Accordingly, $w\approx \Theta \sqrt{20\alpha \varepsilon_{s}}$ to lowest order in Θ. In a like manner to section 4.2, when the distance, $\sim \pi {\dot{\varepsilon }}_{s}/\omega$, the stationary point migrates over a half cycle is comparable to *w*, further decrease of Θ is thwarted by the evolving level curves pattern. This implies that the amplitude will not decrease below a characteristic value $\Theta_{\text{min}}\approx \pi \left|{\dot{\varepsilon }}_{s}/\varepsilon_{s}\right|/40\chi \alpha$. This value depends inversely on *χ* = Ω_⊙_*t*_*T*_. Thus for slower tidal evolution (i.e., larger tidal time constant *t*_*T*_, smaller terrestrial Δ*t*), the libration amplitude can be decreased to smaller values during the initial damped outbound phase.

Once excitation begins at time *τ*_*ex*_, integrating equation (6.9) gives

(6.14) $$\text{ln}\left(\frac{\Theta }{\Theta_{ex}}\right)=\frac{1}{2}\overset{\tau }{\underset{\tau_{ex}}{\int }}\left(\frac{\partial {\dot{\varepsilon }}_{T}}{\partial \varepsilon }-\frac{\partial {\dot{\varepsilon }}_{s}}{\partial \varepsilon }\right)\;d\tau ,$$

where Θ_*ex*_ ≈ Θ_*min*_(*τ*_*ex*_) denotes the amplitude at *τ*_*ex*_. Thus, the amplitude grows as

(6.15) $$\Theta (\tau )=\Theta_{\text{min}}\left(\tau_{ex}\right)\text{exp}\left[\frac{1}{2}\int_{\tau_{ex}}^{\tau }\left(\frac{\partial {\dot{\varepsilon }}_{T}}{\partial \varepsilon }-\frac{\partial {\dot{\varepsilon }}_{s}}{\partial \varepsilon }\right)\;d\tau \right].$$

For a given evolution, one can integrate (6.15) to estimate when Θ → *π*/2 and escape occurs as a function of *A* and the absolute rate of tidal evolution given by *χ*. For *A* ≤ 10, *L*_0_ = 2*L*_EM_, a nonsynchronously rotating Moon, and 1 × 10^6^ ≤ *t*_*T*_ ≤ 2 × 10^7^ (corresponding approximately to 80 < (*Q*/*k*_*T*_) < 1800), escape occurs early when the Earth-Moon system AM has been reduced by only about 8% to 9% relative to its starting value. For *A* = 10, *L*_0_ = 2*L*_EM_, and a synchronously rotating moon (including the effects of permanent figure torques), the change in AM is even less, about 3% to 5%.

## 7. Summary and Discussion

We have examined the tidal evolution of the Sun-Moon evection resonance employing the tidal model developed by Mignard (1980). This has been motivated by the work of Ćuk and Stewart (2012; CS12) who found a large decrease in the Earth-Moon system AM due to this mechanism. Although the direct solar tidal torque on the Earth can drain its spin AM, the loss is very small over the age of the solar system. In contrast, the evection resonance allows the Sun to indirectly drain the Earth’s spin by exerting a torque on the lunar orbit that can then be transmitted to the Earth via the much stronger lunar tidal torque. Initial capture of the Moon into evection is not guaranteed. However, the case has been made that capture is probable given the slow outward tidal evolution rates associated with a fluid-like Earth in the aftermath of a Moon-forming giant impact (Zahnle et al., 2015). If the evection resonance is then maintained, the loss of AM could potentially be very large.

CS12 utilized a tidal model intended to approximate a constant-*Q* model, in which the tide is assumed to form at a fixed angle ahead or behind the line connecting the centers of the tidally interacting objects. In order to avoid discontinuity at the synchronous orbit, they multiplied their tidal torque by a smoothing factor. A detailed analysis of the CS12 tidal model and its differences from a conventional constant-*Q* model (Kaula, 1964) is contained in Wisdom and Tian (2015). They implemented a true constant-*Q* model and found that if the Moon’s tidal parameters are assumed constant with time, the successful cases identified in CS12 remove too much AM. Tian et al. (2017) subsequently demonstrated that tidal heating during the high-eccentricity evolution in evection invoked in CS12 would alter tidal dissipation in the Moon and cause rapid exit from formal resonance with little or no AM drain, again assuming a constant-*Q* tidal model.

In this paper, we adopt the Mignard tidal model as was also utilized by Touma and Wisdom (1998) but apply it to an Earth-Moon system that initially has a much higher AM than its current value, as considered in CS12. Our findings include the following:

1. AM is drained from the Earth-Moon system while the evection resonance is maintained. In the limiting case of a moon that remained continually in resonance, evection would drive the system to a co-synchronous end state, *s* = *s*_*m*_ = *n*, with a final AM independent of the system’s initial AM. For the Earth-Moon system this limiting state was never reached, implying that either the Moon was never captured into evection or that it escaped from resonance. In the latter case, the timing of escape determines the degree of AM modification due to formal evection, with increased AM drain as the time spent in resonance lengthens.
2. During resonance, the Moon’s longitude of perigee librates about the stationary state angle (which is approximately ±90° from the Earth-Sun line). Escape from resonance requires the resonant trajectory to cross the separatrix boundary, which can occur if the libration amplitude, Θ, approaches *π*/2. Tidal evolution causes libration excitation and/or damping if there is a variation in tidal strength over a libration cycle.
3. For Mignard tides, resonant libration is damped or minimally excited during most of the Moon’s initial outward expansion in resonance. However, as the Moon approaches the “stall” point (after which its orbit contracts), libration amplitude excitation increases and remains positive throughout the rest of the evolution. This is true across a wide range of tidal parameters and for either a nonsynchronously rotating moon with no permanent figure torques or a synchronously rotating, triaxial moon.
4. We estimate that libration excitation leads to escape from resonance early in the evolution, resulting in ≤10% AM loss for an Earth-Moon system with an initial AM that is roughly twice that of the current Earth-Moon. This is similar to early resonance escape seen in Touma and Wisdom (1998) with Mignard tides for lower AM systems.

We conclude that with Mignard tides, formal evection resonance does not appear capable of reconciling high-AM giant impact models (Canup, 2012; Ćuk & Stewart, 2012) with the current Earth-Moon system. This result augments those of Wisdom and Tian (2015) and Tian et al. (2017), who conclude that formal evection is unsuccessful in reproducing the Earth-Moon AM for constant-*Q* tides.

Alternatively, appropriate AM removal to accommodate a high-AM Moon-forming giant impact could result from effects other than formal libration in evection. With constant-*Q* tides, Wisdom and Tian (2015) identi-fied an evection-related limit cycle in which large amounts of AM can be extracted from the Earth-Moon even though the Moon is not librating within resonance; a broadly similar “quasi-resonance” was seen in preliminary integrations using the Mignard model by Ward and Canup (2013) and Rufu and Canup (2019). Such effects are not accessible with the methods here. It has also been proposed (Ćuk et al., 2016) that an entirely different mechanism could have reduced the early Earth-Moon AM, involving an initial Earth with a very high obliquity and a Laplace plane instability as the lunar orbit expands. However, the range of successful parameters for this mechanism remains unclear.

The analytic developments here include simplifications, notably co-planar dynamics, an evolution description limited to fourth order in eccentricity, and an assumption of small libration amplitude when assessing how the amplitude varies with time. Ultimately, integration of the system’s full evolution in *a*, *s*, *s*_*m*_, *e*, and θ is needed to assess the behavior of evection in the context of the Mignard tidal model, which will be a topic of a subsequent paper. Additional effects not considered here include the potential time-dependence of the tidal parameters during evolution in evection, and the potential for spin-orbit resonances in the Moon’s rotation state that differ from the non-synchronous or synchronous rotations considered here.

## Supplementary Material

Supplementary Information

## Figures and Tables

**Table 1:** Some Variable Definitions

Semimajor axis, mean motion, and eccentricity of moon	*a*, *n*, *e*
Earth mass and radius	*M*, *R*
Lunar mass and radius	*m*, *R*_*m*_
Mass ratio *m/M*	*μ*
Earth spin rate, lunar spin rate	*s*, *s*_*m*_
Angular momentum of Earth-Moon system	*L*_EM_
Angular momentum of lunar orbit	*L*_*orb*_
Measure of the strength ratio of lunar to Earth tides	*A*
Maximum principal moments of inertia of Earth, Moon	*C*, *C*_*m*_
Ratio of principal moments *C*_*m*_/*C*	*κ*
Gyration constant for Earth	*λ*
Ratio of *μ*/*λ*	*γ*
Circumterrestrial orbital frequency at *R*	Ω_⊕_
Earth’s orbital frequency about the Sun	Ω_⊙_
Tidal lag times, lag angle, and Love numbers for Earth, Moon	Δ*t*, Δ*t*_*m*_, *δ*, *k*_*T*_, *k*_*m*_
Tidal evolution time constant	*t*_*T*_
Normalized tidal evolution time	*χ* = Ω_⊙_*t*_*T*_
Libration amplitude	Θ

**Table 2:** Tidal Polynomials and their Derivatives

${\tilde{f}}_{1}(\varepsilon )=1+\frac{15}{2}\epsilon +\frac{45}{8}\varepsilon^{2}+\frac{5}{16}\varepsilon^{3}$	; $\frac{\partial {\tilde{f}}_{1}(\varepsilon )}{\partial \varepsilon }=\frac{15}{2}+\frac{45}{4}\varepsilon +\frac{15}{16}\varepsilon^{2}$
${\tilde{f}}_{2}(\varepsilon )=1+\frac{31}{2}\epsilon +\frac{255}{8}\epsilon^{2}+\frac{185}{16}\epsilon^{3}+\frac{25}{64}\epsilon^{4}$	; $\frac{\partial {\tilde{f}}_{2}(\varepsilon )}{\partial \varepsilon }=\frac{31}{2}+\frac{255}{4}\varepsilon +\frac{555}{16}\varepsilon^{2}+\frac{25}{16}\varepsilon^{3}$
${\bar{g}}_{1}(\varepsilon )=\frac{11}{2}+\frac{33}{4}\varepsilon +\frac{11}{16}\varepsilon^{2}$	; $\frac{\partial {\tilde{g}}_{1}(\varepsilon )}{\partial \varepsilon }=\frac{33}{4}+\frac{11}{8}\varepsilon$
${\tilde{g}}_{2}(\varepsilon )=9+\frac{135}{4}\varepsilon +\frac{135}{8}\varepsilon^{2}+\frac{45}{64}\varepsilon^{3}$	; $\frac{\partial {\tilde{g}}_{2}(\varepsilon )}{\partial \varepsilon }=\frac{135}{4}+\frac{135}{4}\varepsilon +\frac{135}{64}\varepsilon^{2}$

## Key Points:

- Capture of the Moon into the evection resonance with the Sun transfers angular momentum from the Earth-Moon to Earth’s heliocentric orbit
- Using the Mignard tidal model, we find that escape from evection occurs early with minimal angular momentum loss from the Earth-Moon
- Processes beyond formal evection resonance are needed to reconcile a high-angular momentum giant impact with the current Earth-Moon
