# Supplementary material for: Analytical Model for the Tidal Evolution of the Evection Resonance and the Timing of Resonance Escape
Source: J Geophys Res Planets. Author manuscript; Available in PMC 2020 Oct 9. (PMC7545365; doi:10.1029/2019je006266)
Supplement: Supplementary Information [file NIHMS1623081-supplement-Supplementary_Information.pdf]

AGU PUBLICATIONS

JGR-Planets

Supporting Information for

Analytical Model for the Tidal Evolution of the Evection Resonance and the Timing of Resonance Escape

William R. Ward, Robin M. Canup, and Raluca Rufu

Planetary Science Directorate Southwest Research Institute; Boulder, CO 80302

Contents of this file

Appendix A to E
Figures A1 to A3

Appendix A - Jacobi Constant

The lunar equation of motion with Earth's oblateness and Sun's influence treated as a disturbing potential, $\mathcal{R} = -(\Phi_\oplus + \Phi_\odot)$, in the coordinate system centered on Earth is:

$$\ddot{\vec{r}} = \nabla(U + \mathcal{R}) \quad \text{(A1)}$$

where $U \approx GM/r$ is the two-body Earth-Moon potential. Here the Moon's mass is ignored, and the lunar inclination and terrestrial obliquity are assumed negligible ($\ddot{z} = 0$), hence:

$$\ddot{x} = \frac{\partial}{\partial x}(U + \mathcal{R}) = GM\frac{\partial}{\partial x}\left(\frac{1}{\sqrt{x^2+y^2}}\right) + \frac{\partial \mathcal{R}}{\partial x} \quad \text{(A2a,b)}$$

$$\ddot{y} = \frac{\partial}{\partial y}(U + \mathcal{R}) = GM\frac{\partial}{\partial y}\left(\frac{1}{\sqrt{x^2+y^2}}\right) + \frac{\partial \mathcal{R}}{\partial y}$$

This can be rearranged to (Brouwer and Clemence, 1961):

$$\ddot{x} + \frac{GMx}{r^3} = \frac{\partial \mathcal{R}}{\partial x} \quad \text{(A3a, b)}$$

$$\ddot{y} + \frac{GMy}{r^3} = \frac{\partial \mathcal{R}}{\partial y}$$

We switch to a rotating coordinate system $(\mathcal{X}, \mathcal{Y})$, where the $\mathcal{X}$ axis is aligned along the Earth-Sun line ($\vec{r}'$) and the system rotates with an angular velocity of $\Omega_\odot$ (assuming that Earth's orbit around the Sun is circular), with (Murray and Dermott, 1999) :

$$x = \mathcal{X}\cos\Omega_\odot t - \mathcal{Y}\sin\Omega_\odot t \qquad \text{(A4a,b,c,d)}$$

$$y = \mathcal{X}\sin\Omega_\odot t + \mathcal{Y}\cos\Omega_\odot t$$

$$\ddot{x} = \left(\ddot{\mathcal{X}} - 2\Omega_\odot\dot{\mathcal{Y}} - \Omega_\odot^2\mathcal{X}\right)\cos\Omega_\odot t - \left(\ddot{\mathcal{Y}} + 2\Omega_\odot\dot{\mathcal{X}} - \Omega_\odot^2\mathcal{Y}\right)\sin\Omega_\odot t$$

$$\ddot{y} = \left(\ddot{\mathcal{X}} - 2\Omega_\odot\dot{\mathcal{Y}} - \Omega_\odot^2\mathcal{X}\right)\sin\Omega_\odot t + (\ddot{\mathcal{Y}} + 2\Omega_\odot\dot{\mathcal{X}} - \Omega_\odot^2\mathcal{Y})\cos\Omega_\odot t$$

Substituting these relations into the equations of motion, multiplying (A.3a) by $\cos\Omega_\odot t$, and (A.3b) by $\sin\Omega_\odot t$, and adding the results gives

$$\ddot{\mathcal{X}} - 2\Omega_\odot\mathcal{Y} - \Omega_\odot^2\mathcal{X} + \frac{GM\mathcal{X}}{r^3} = \frac{\partial\mathcal{R}}{\partial\mathcal{X}} \qquad \text{(A5)}$$

where the LHS of the equation is given by the chain rule: $\frac{\partial\mathcal{R}}{\partial\mathcal{X}} = \frac{\partial\mathcal{R}}{\partial x}\frac{\partial x}{\partial\mathcal{X}} + \frac{\partial\mathcal{R}}{\partial y}\frac{\partial y}{\partial\mathcal{X}} = \frac{\partial\mathcal{R}}{\partial x}\cos\Omega_\odot t + \frac{\partial\mathcal{R}}{\partial y}\sin\Omega_\odot t$. Similarly, multiplying (A.3a) by $-\sin\Omega_\odot t$, and (A.3b) by $\cos\Omega_\odot t$, and adding the results:

$$\ddot{\mathcal{Y}} + 2\Omega_\odot\dot{\mathcal{X}} - \Omega_\odot^2\mathcal{Y} + \frac{GM}{r^3} = \frac{\partial\mathcal{R}}{\partial\mathcal{Y}} \qquad \text{(A6)}$$

where the LHS of the equation is given by the chain rule: $\frac{\partial\mathcal{R}}{\partial\mathcal{Y}} = \frac{\partial\mathcal{R}}{\partial x}\frac{\partial x}{\partial\mathcal{Y}} + \frac{\partial\mathcal{R}}{\partial y}\frac{\partial y}{\partial\mathcal{Y}} = -\frac{\partial\mathcal{R}}{\partial x}\sin\Omega_\odot t + \frac{\partial\mathcal{R}}{\partial y}\cos\Omega_\odot t$. The last two expressions can be simplified by (Brouwer and Clemence, 1961):

$$\ddot{\mathcal{X}} - 2\Omega_\odot\dot{\mathcal{Y}} = \frac{\partial F}{\partial\mathcal{X}} \qquad \text{(A7a, b)}$$

$$\ddot{\mathcal{Y}} + 2\Omega_\odot\dot{\mathcal{X}} = \frac{\partial F}{\partial\mathcal{Y}}$$

where $F \equiv \frac{GM}{r} + \frac{\Omega^2}{2}(\mathcal{X}^2 + \mathcal{Y}^2) + \mathcal{R}$.

To get the Jacobi integral, we multiply (A.7a) by $\dot{\mathcal{X}}$, (A.7b) by $\dot{\mathcal{Y}}$ and add them:

$$\ddot{\mathcal{X}}\dot{\mathcal{X}} + \ddot{\mathcal{Y}}\dot{\mathcal{Y}} = \frac{\partial F}{\partial\mathcal{X}}\dot{\mathcal{X}} + \frac{\partial F}{\partial\mathcal{Y}}\dot{\mathcal{Y}} \qquad \text{(A8)}$$

Integrating the last expression:

$$\frac{1}{2}\dot{\mathcal{X}}^2 + \frac{1}{2}\dot{\mathcal{Y}}^2 = F + \frac{J}{m} \qquad \text{(A9)}$$

where J is the modified Jacobi constant (in energy units).

$$\frac{1}{2}\left(\dot{\mathcal{X}}^2 + \dot{\mathcal{Y}}^2\right) - \frac{GM}{r} - \frac{\Omega_\odot^2}{2}(\mathcal{X}^2 + \mathcal{Y}^2) - \mathcal{R} = \frac{J}{m} \qquad \text{(A10)}$$

We return to the non-rotating frame, centered on Earth, to express the Jacobi constant in terms of the Moon's a and e. We use the relation:

$$\dot{\mathcal{X}}^2 + \dot{\mathcal{Y}}^2 = \dot{x}^2 + \dot{y}^2 + \Omega_\odot^2(x^2 + y^2) + 2\Omega_\odot(\dot{x}y - \dot{y}x) \qquad \text{(A11)}$$

(Note that $\mathcal{X}^2 + \mathcal{Y}^2 = x^2 + y^2$, since distances are invariant under rotation transformations) to yield

$$\frac{1}{2}(\dot{x}^2 + \dot{y}^2) + \Omega_\odot(\dot{x}y - \dot{y}x) - \frac{GM}{r} - \mathcal{R} = \frac{J}{m} \qquad \text{(A12)}$$

The kinetic energy can be replaced by (Murray and Dermott, 1999),

$$\tfrac{1}{2}(\dot{x}^2 + \dot{y}^2) = GM\left(\tfrac{1}{r} - \tfrac{1}{2a}\right) \qquad \text{(A13)}$$

and we set $\dot{y}x - \dot{x}y = \vec{r}\cdot\vec{v} = L_{orb}/m = \sqrt{GMa(1-e^2)}$. Substituting these into (A12) gives eqn. (2.5) in the main text,

$$J = m\left[-\frac{GM}{2a} - \mathcal{R} - \Omega_\odot\sqrt{GMa(1-e^2)}\right] .$$

Appendix B - Stationary States

Tidal free states

Denoting $\tilde{\alpha} \equiv 2\alpha(1 - 5\cos 2\theta)$, eqn. (2.15) is rearranged as, $\varepsilon = 1 - \eta[1 - \tilde{\alpha}(1-\varepsilon)^{1/2}]^{-1/2}$. In the limit $\tilde{\alpha} \to 0, \varepsilon \to 1 - \eta$. For small $\tilde{\alpha}$, we write $\varepsilon = 1 - \eta + \Delta\varepsilon$, to find

$$\Delta\varepsilon = \eta\{1 - [1 - \tilde{\alpha}(\eta - \Delta\varepsilon)^{1/2}]^{-1/2}\} \qquad \text{(B1)}$$

To second order accuracy in α, the RHS is explicitly expanded to second order in $\tilde{\alpha}$,

$$\Delta\varepsilon \approx -\eta\{\tfrac{1}{2}\tilde{\alpha}(\eta - \Delta\varepsilon)^{1/2} + \tfrac{3}{8}\tilde{\alpha}^2(\eta - \Delta\varepsilon)] . \qquad \text{(B2)}$$

Assuming $\Delta\varepsilon \sim \mathcal{O}(\alpha)$ as well, $(\eta - \Delta\varepsilon)^{1/2} \approx \eta^{1/2}(1 - \Delta\varepsilon/2\,\eta)$. This leads to

$$\Delta\varepsilon \approx -\tfrac{1}{2}\tilde{\alpha}\eta^{3/2}(1 + \tfrac{3}{4}\tilde{\alpha}\eta^{1/2})/(1 - \tfrac{1}{4}\tilde{\alpha}\eta^{1/2}) \approx -\tfrac{1}{2}\tilde{\alpha}\eta^{3/2}(1 + \tilde{\alpha}\eta^{1/2}), \qquad \text{(B3)}$$

and accordingly, $\varepsilon \approx 1 - \eta - \tilde{\alpha}\eta^{3/2}/2 - \tilde{\alpha}^2\eta^2/2$. To lowest order in α, this reduces to $\varepsilon \approx 1 - \eta - \alpha(1 - 5\cos 2\theta)\eta^{3/2}$. Solution of this equation at θ = 0, π yields the *y*-axis stationary point value, $\varepsilon_s \approx 1 - \eta + 4\alpha\eta^{3/2}$, while the unstable stationary points at $\theta = \pm$ π/2 on the *x*-axis are located at $\varepsilon_{sx} \approx 1 - \eta - 6\alpha\eta^{3/2}$. Their average value is $\varepsilon_* \approx 1 - \eta - \alpha\eta^{3/2}$.

We further simply by neglecting terms of order $\alpha\varepsilon$; combining the above expression for ε_* with $\eta \approx (1-\varepsilon)$ from eqn. (2.15) then gives $\varepsilon_* \approx 1 - \eta - \alpha(1-\varepsilon)^{3/2} \approx 1 - \eta - \alpha$, $\varepsilon_s \approx 1 - \eta + 4\alpha = \varepsilon_* + 5\alpha$, and $\varepsilon_{sx} \approx 1 - \eta - 6\alpha = \varepsilon_* - 5\alpha$.

Tidal states

Because tides displace the stationary angle off the y-axis, there is a net average solar torque. The solar torque at the stationary point from eqn. (3.2b) becomes

$$\mathrm{T}' = 10\gamma\chi a'^{1/2}\alpha\varepsilon_s \sin 2\theta_s = (\gamma/2)a'^{1/2}(\dot{\varepsilon}_T - \dot{\varepsilon}) \ , \qquad \text{(B4)}$$

while the rate at which the system angular momentum must change is $\dot{L}_{orb} - \dot{L}_{orb,T}$, *i.e.*,$(\gamma/2)a'^{1/2}(\dot{\varepsilon}_T - \dot{\varepsilon}_s)/(1-\varepsilon_s)^{1/2}$. These agree if eqn. (5.8) is used to evaluate $\sin 2\theta_s = (\dot{\varepsilon}_T - \dot{\varepsilon}_s)/20\chi\alpha\varepsilon_s$. However, since the extreme value of $\sin 2\theta_s \approx (\dot{\varepsilon}_T - \dot{\varepsilon}_*)/20\chi\alpha\varepsilon_* \to -1$, the strongest possible torque is $\mathrm{T}'_{max} = -10\chi\alpha\gamma a'^{1/2}\varepsilon_*$. Accordingly, the resonance could not be maintained if

$$\chi \equiv \Omega_\odot t_T < -(\dot{\varepsilon}_T - \dot{\varepsilon}_s)/20\alpha\varepsilon_*(1-\varepsilon_*) \equiv \chi_{crit} \qquad \text{(B5)}$$

Appendix C - Mignard Tidal Model

Mignard first derives the force due to a second-order tidal distortion raised on the Earth by the Moon in the vector form,

$$F = -3k_T \frac{Gm^2R^5}{r^{10}} \Delta t[2(\boldsymbol{r}\cdot\boldsymbol{v})\boldsymbol{r} + r^2(\boldsymbol{r}\times\boldsymbol{s} + \boldsymbol{v})] \qquad \text{(C1)}$$

Where k_T is the tidal Love number for the Earth, vectors $\boldsymbol{r}, \boldsymbol{v}$ are the position and velocity of the Moon of mass m, and $\boldsymbol{s}$ is the Earth's spin vector, which, for simplicity, we will assume is perpendicular to the lunar orbit plane. The radial, F_r, and tangential, F_θ, force components are then substituted into Gauss' form of the Lagrange equations (*e.g.*, Brouwer and Clemence, 1961),

$$\frac{da}{dt} = \frac{2}{mn(1-e^2)^{1/2}}\left[F_r e \sin\theta + F_\theta \frac{p}{r}\right];\ \frac{de}{dt} = \frac{(1-e^2)^{1/2}}{man}\left[F_r \sin\theta + F_\theta \frac{1}{e}\left(\frac{p}{r} - \frac{r}{a}\right)\right] \qquad \text{(C2a,b)}$$

where $p \equiv a(1-e^2)$ and the rates are then averaged over an orbit to give the tidal changes in semi-major-axis and eccentricity.

Appendix D - Permanent Figure Torque

Consider a Moon with principal moments of inertia $C_m \geq B_m \geq A_m$, where C_m is the moment about its spin axis, assumed to be normal to its orbit plane, and A_m is the moment about the Moon's long axis. The instantaneous value of the permanent figure (*pf*) torque is given by Danby (1992; see also Murray and Dermott, 1999),

$$T_{pf} = -\frac{3}{2}(B_m - A_m)(GM/r^3)\, sin\, 2\psi = C_m \frac{ds_{m,pf}}{dt} \qquad \text{(D1)}$$

where *r* is the Earth-Moon distance, ψ is the angle between the long axis of the Moon and the Earth-Moon line, *i.e*, $\psi = \vartheta - f$, where ϑ is the angular position of the Moon's long axis with respect to the perigee, ϖ, and f is the true anomaly (*e.g.*, Goldreich and Peale, 1966a,b). We set $\vartheta = s_m t + \psi_o$, which for synchronous rotation is $\vartheta = nt + \psi_o$, where ψ_o is the value of ψ at perigee. If T_{pf} is then averaged over an orbit, one obtains (*e.g.*, Goldreich and Peale, 1966a,b),

$$\langle T_{pf}\rangle = -\frac{3}{2}n^2(B_m - A_m)H(\varepsilon)\sin 2\psi_o \qquad \text{(D2)}$$

where $H(\varepsilon) = 1 - 5\varepsilon/2 + 13\,\varepsilon^2/16$ is a so-called Hansen polynomial. This torque leads to further contributions to semi-major axis and eccentricity variations, $\dot{a}'_{pf}$ and $\dot{\varepsilon}_{pf}$.

Analogous to eqn. (4.2), conservation of angular momentum requires

$$\dot{s}'_{m,pf} = -\frac{\gamma}{2\kappa} a'^{1/2}(1-\varepsilon)^{1/2}\left(\frac{\dot{a}'_{pf}}{a'} - \frac{\dot{\varepsilon}_{pf}}{1-\varepsilon}\right). \quad \text{(D3)}$$

This must (nearly) balance the tidal torque to ensure synchronous stability so that $\dot{s}'_m + \dot{s}'_{m,pf} = \dot{n}/\Omega_\oplus$, and the off-set angle ψ_o adopts the value needed to accomplish this.

A major difference between a torque on the permanent figure of the Moon and a torque on a tidal distortion is that the former is not accompanied by energy dissipation due to planetary flexing. Accordingly, the combination of orbital energy and spin energy of the Moon is also conserved under its action, *i.e.*, $d(\kappa\lambda s'^2_m/2 - \mu/2a')/d\tau|_{PF} = 0$. Taking the derivatives and rearranging yields an additional condition,

$$\dot{s}'_{m,pf} = -\frac{\gamma}{2\kappa}\frac{\dot{a}'_{pf}}{s'_m a'^2} = -\frac{\gamma}{2\kappa} a'^{1/2}\frac{\dot{a}'_{pf}}{a'} \quad \text{(D4)}$$

where the final expression sets $s'_m = n/\Omega_\oplus = a'^{-3/2}$.

Approximating $\dot{s}'_m + \dot{s}'_{m,pf} \approx 0$ due to the smallness of $\dot{n}$ compared to either spin acceleration, we conclude that

$$\left(\frac{\dot{a}'_{pf}}{a'} - \frac{\dot{\varepsilon}_{pf}}{1-\varepsilon}\right) \approx -\left(\frac{\dot{a}'_m}{a'} - \frac{\dot{\varepsilon}_m}{1-\varepsilon}\right) \quad \text{(D5)}$$

However, one cannot simply assume equal but opposite values for $\dot{a}'_{pf} = -\dot{a}'_m$ and $\dot{\varepsilon}_{pf} = -\dot{\varepsilon}_m$, because the permanent figure torque may partition its changes in a and ε differently than do tides. From eqns. (D3) and (D4) we get

$$\frac{\left[1-(1-\varepsilon)^{1/2}\right]}{(1-\varepsilon)^{1/2}}\frac{\dot{a}'_{pf}}{a'} = -\frac{\dot{\varepsilon}_{pf}}{1-\varepsilon} \quad \text{(D6)}$$

Using this to eliminate either $\dot{a}'_{pf}$ or $\dot{\varepsilon}_{pf}$ in eqn. (D5) leads to,

$$\frac{\dot{a}'_{pf}}{a'} = -f_{pf}\left(\frac{\dot{a}'_m}{a'} - \frac{\dot{\varepsilon}_m}{1-\varepsilon}\right) \;\; ; \; \dot{\varepsilon}_{pf} = g_{pf}\left(\frac{\dot{a}'_m}{a'} - \frac{\dot{\varepsilon}_m}{1-\varepsilon}\right) \quad \text{(D7a,b)}$$

where $f_{pf} \equiv (1-\varepsilon)^{1/2}$ and $g_{pf} = (1-\varepsilon)\left[1-(1-\varepsilon)^{1/2}\right]$. The total change rates for a' and ε due to both tides and T_{pf} for a Moon in synchronous rotation is then

$$\frac{\dot{a}'}{a'} = \frac{\dot{a}'_\oplus}{a'} + \left(1-f_{pf}\right)\frac{\dot{a}'_m}{a'} + f_{pf}\frac{\dot{\varepsilon}_m}{1-\varepsilon} \quad \text{(D8)}$$

$$\dot{\varepsilon}_T = \dot{\varepsilon}_\oplus + \left(1-\frac{g_{pf}}{1-\varepsilon}\right)\dot{\varepsilon}_m + g_{pf}\frac{\dot{a}'_m}{a'}\;, \quad \text{(D9)}$$

where $s'_m a'^{3/2} = 1$, which in combination with (4.9a,b) gives

$$\dot{a}'_m/a' = A[f_1(\varepsilon) - f_2(\varepsilon)]/a'^8 \quad ; \quad \dot{\varepsilon}_m = A\varepsilon[g_1(\varepsilon) - g_2(\varepsilon)]/a'^8 \quad \text{(D10a,b)}$$

The above rates are valid so long as synchronous rotation can be maintained. However, $|\sin 2\psi_o|$ has a maximum value of unity, and so from eqns. (4.2) and (D2) there is a minimum value required for $(B_m - A_m)/C_m$,

$$\frac{(B_m - A_m)}{C_m} > \left|\frac{\gamma}{3\kappa}\left(\frac{a'^{7/2}}{\Omega_\oplus \tau_T}\right)\frac{(1-\varepsilon)^{1/2}}{H(\varepsilon)}\left(\frac{\dot{a}'_m}{a'} - \frac{\dot{\varepsilon}_m}{1-\varepsilon}\right)\right|$$

$$= \left|\frac{\gamma}{3\kappa}\left(\frac{A}{\Omega_\oplus \tau_T}\right)\frac{1}{a'^{9/2}}\frac{(1-\varepsilon)^{1/2}}{H(\varepsilon)}\left[f_1 - f_2 - \frac{\varepsilon}{1-\varepsilon}(g_1 - g_2)\right]\right| \quad \text{(D11a)}$$

where the final expression sets $s'_m a'^{3/2} = 1$. This criterion reads

$$\frac{(B_m - A_m)}{C_m} > 4 \times 10^{-4}\left(\frac{k_m \Delta t_m}{4 \text{ min}}\right)\frac{(1-\varepsilon)^{1/2}}{H(\varepsilon)}\left[f_1 - f_2 - \frac{\varepsilon}{1-\varepsilon}(g_1 - g_2)\right]\left(\frac{7}{a'}\right)^{9/2} \quad \text{(D11b)}$$

where $k_m \Delta t_m \approx 4$ min for the current Moon (Williams and Boggs, 2015). If violated, the synchronous lock is broken.

The above estimate considers whether the permanent figure torque is sufficient to maintain synchronous rotation against the competing tidal torque. Goldreich (1966)

considered an initial rotation faster than n, and found that this rate would decrease, librate about synchronous rotation, and ultimately damp to the synchronous state if

$$\frac{(B_m - A_m)}{C_m} \gtrsim 7.5\pi^2\varepsilon^2 \tag{D12}$$

For a shape similar to that of the current Moon, with $(B_m - A_m)/C_m = 2.28 \times 10^{-4}$, eqn. (D11b) implies that synchronous lock could be maintained at the time the Moon encounters evection (*i.e.*, $a' \sim 7$) for an initially low eccentricity ($\varepsilon < 0.095$), but that non-synchronous rotation would ensue as e became large. Eqn. (D12) implies that the $(B_m - A_m)/C_m$ of the current Moon would be sufficient to establish synchronous rotation for $\varepsilon < 0.0018$. Of course, the current $(B_m - A_m)/C_m$ value may not have pertained to the early Moon, and so it is prudent to consider both synchronous and non-synchronous cases.

For the case of a non-synchronously rotating Moon without permanent figure torques, eqns. (6.10) and (6.12) in the main text provide the partial derivatives needed to evaluate whether the libration amplitude grows or damps. Analogous expressions can be developed for synchronous lunar rotation maintained by a permanent figure torque, with $\partial(\dot{\varepsilon}_T)/\partial\varepsilon$ replaced by $\partial(\dot{\varepsilon}_T + \dot{\varepsilon}_{pf})/\partial\varepsilon$, and $\partial(\dot{a}'/a')/\partial\varepsilon$ replaced by $\partial(\{\dot{a}' + \dot{a}'_{pf}\}/a')/\partial\varepsilon$, with $\dot{\varepsilon}_{pf}$ and $\dot{a}'_{pf}$ given in (D7). These are

$$\frac{a'^8}{A}\frac{\partial\dot{\varepsilon}_{pf}}{\partial\varepsilon} = (f_1 - f_2)\frac{\partial g_{pf}}{\partial\varepsilon} + \left(\frac{\partial f_1}{\partial\varepsilon} - \frac{\partial f_2}{\partial\varepsilon}\right) g_{pf}$$
$$-(g_1 - g_2)\left(\frac{\partial g_{pf}}{\partial\varepsilon}\frac{\varepsilon}{1-\varepsilon} + \frac{\varepsilon g_{pf}}{(1-\varepsilon)^2} + \frac{g_{pf}}{1-\varepsilon}\right) - \left(\frac{\partial g_1}{\partial\varepsilon} - \frac{\partial g_2}{\partial\varepsilon}\right)\frac{\varepsilon g_{pf}}{1-\varepsilon} \tag{D13}$$

and

$$\frac{a'^8}{A}\frac{\partial}{\partial\varepsilon}\left(\frac{\dot{a}'_{pf}}{a'}\right) = \frac{1}{(1-\varepsilon)^{\frac{1}{2}}} \tag{D14}$$
$$\left[\frac{f_1 - f_2}{2} + (g_1 - g_2)\left(1 + \frac{\varepsilon}{2(1-\varepsilon)}\right) + \varepsilon\left(\frac{\partial g_1}{\partial\varepsilon} - \frac{\partial g_2}{\partial\varepsilon}\right) + \left(\frac{\partial f_2}{\partial\varepsilon} - \frac{\partial f_1}{\partial\varepsilon}\right)(1-\varepsilon)\right],$$

where $\frac{\partial g_{pf}}{\partial\varepsilon} = \frac{3}{2}(1-\varepsilon)^{1/2} - 1$.

Appendix E - Additional Zero Libration Evolutions

Here we show additional zero-libration evolutions as considered in Section 5. Note that in these and the other evolutions in the main text we ignore the potential for tidal disruption when the lunar perigee is interior to the Roche limit, which can occur for low A cases.

Figure A1 displays tracks for $A = 10$ with different starting values of the system angular momentum, L'_0, corresponding to varied initial Earth spin rates, s'_0, following a lunar forming impact. Changing L'_0 alters the encounter distance for the resonance as in Figure 4. For lower L'_0, the resonance occurs closer to the Earth and the stall in the Moon's orbital expansion occurs at smaller a'_c and ε_c . However, all cases eventually converge on the same end state in the limiting case that the Moon remains in resonance throughout its whole evolution (which as we show in Section 6 is unlikely to occur, as much earlier resonance escape is predicted). Accordingly, the higher the starting L'_0, the greater the angular momentum decay, $\Delta L' = L'_0 - L'_f$, and evolutionary tracks for high L'_0 are reminiscent of those shown in CS12.

Figure A2 compares evolutionary tracks with $L_0 = 2L_{EM}$ for other values of A. As A increases, the stationary state eccentricity is suppressed by progressively stronger lunar tides. This in turn weakens the tidal torque (due to the larger lunar periapsis), prolonging the evolutionary time scale. Figure A3 displays a synchronous evolution with $A = 10$, $L_0 = 2\,L_{EM}$ contrasted to the non-synchronous evolution shown in Figure 4 in the main text, shown in grey. Here we have set $s'_m a'^{3/2} = 1$, and modified the expressions for tidal changes in a and ε to include the permanent figure torques as in eqns. (D8) and (D9). The non-synchronous track acquires higher maximum values for a and ε but these then decrease somewhat more rapidly than in the synchronous case.

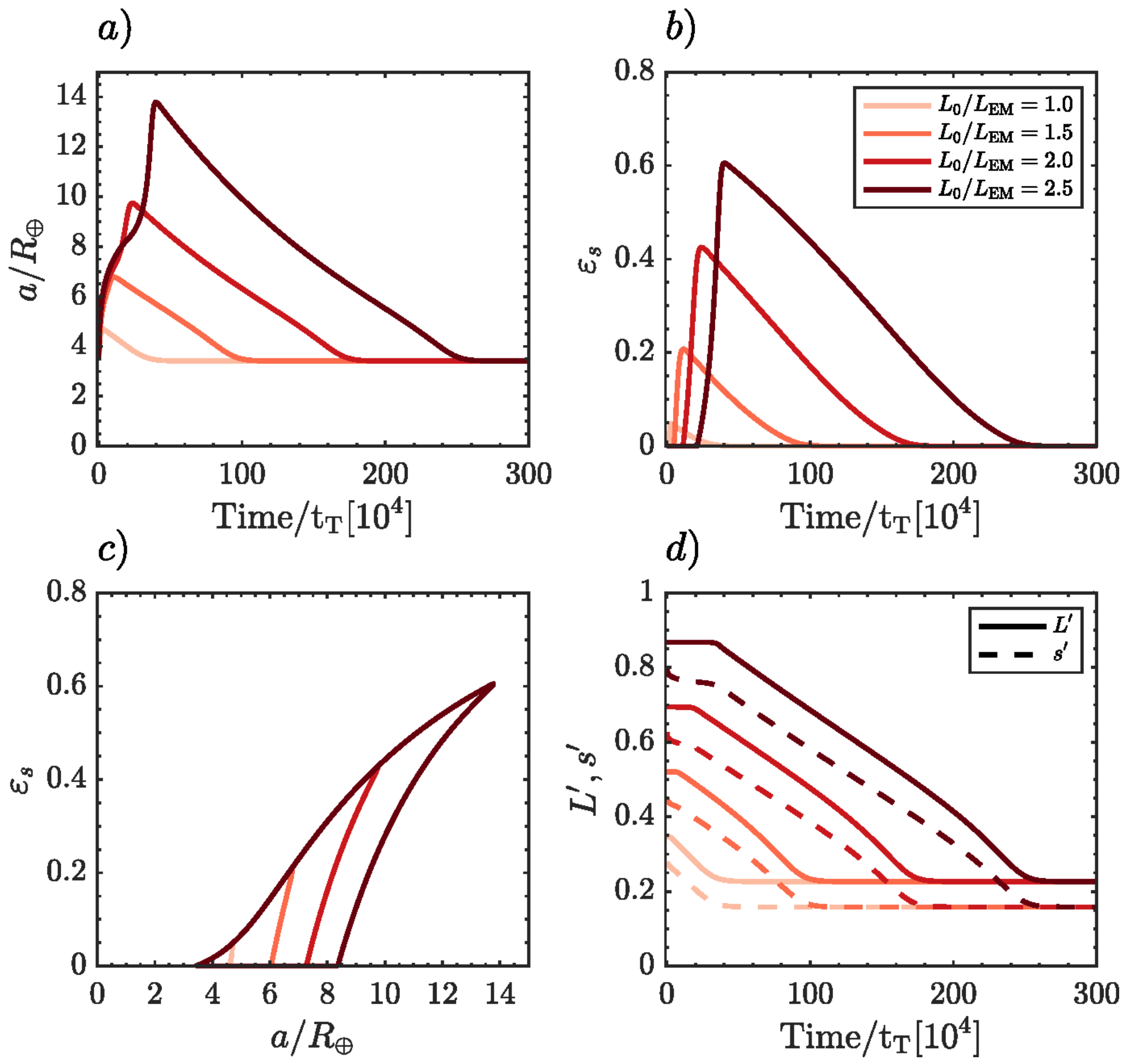

Figure A1. System evolution with A =10 for various values of $\boldsymbol{L_o}$, assuming a Moon in non-synchronous rotation.

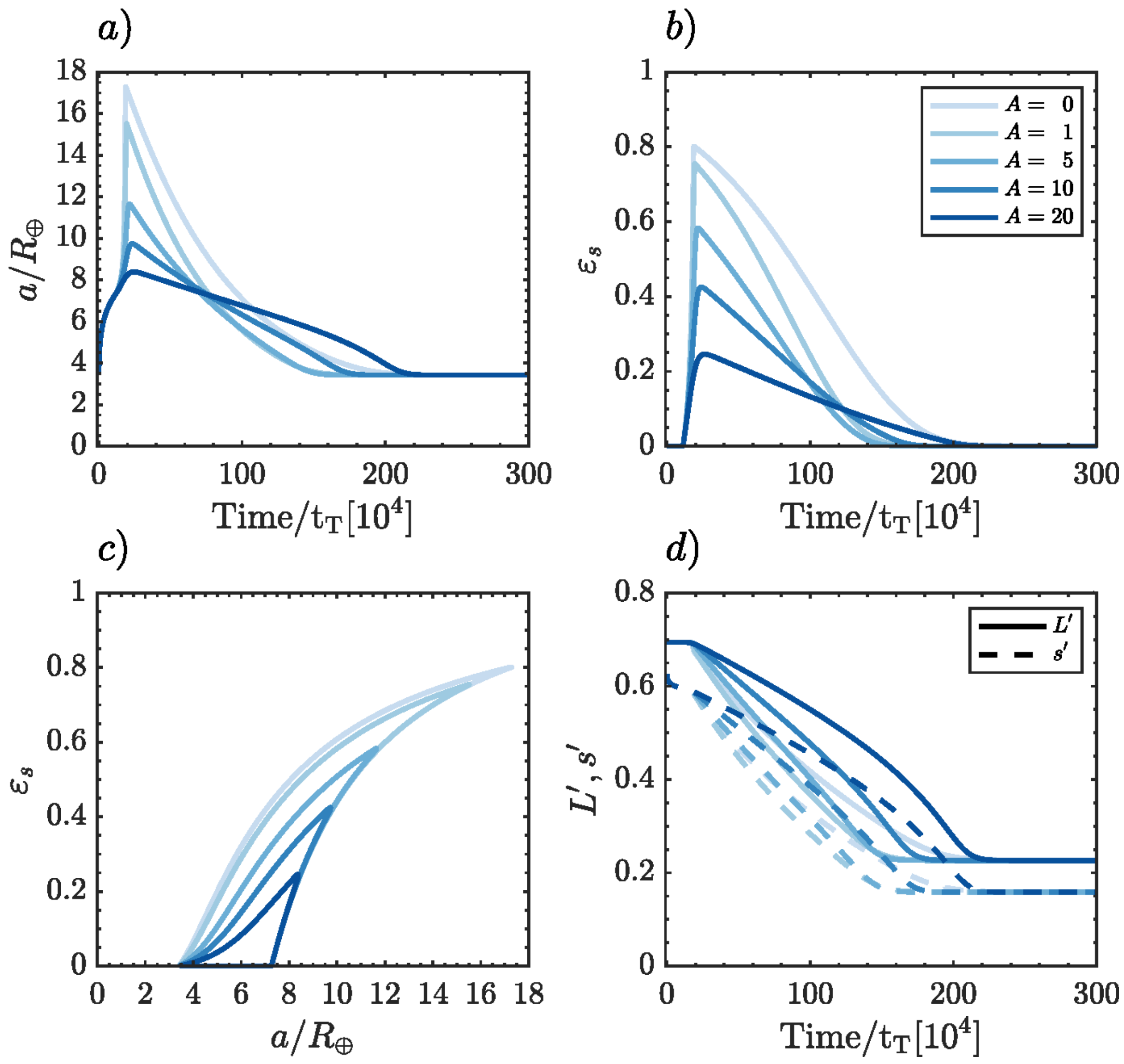

Fig. A2. System evolution for various values of A with $L_o = 2L_{EM}$, assuming a Moon in non-synchronous rotation.

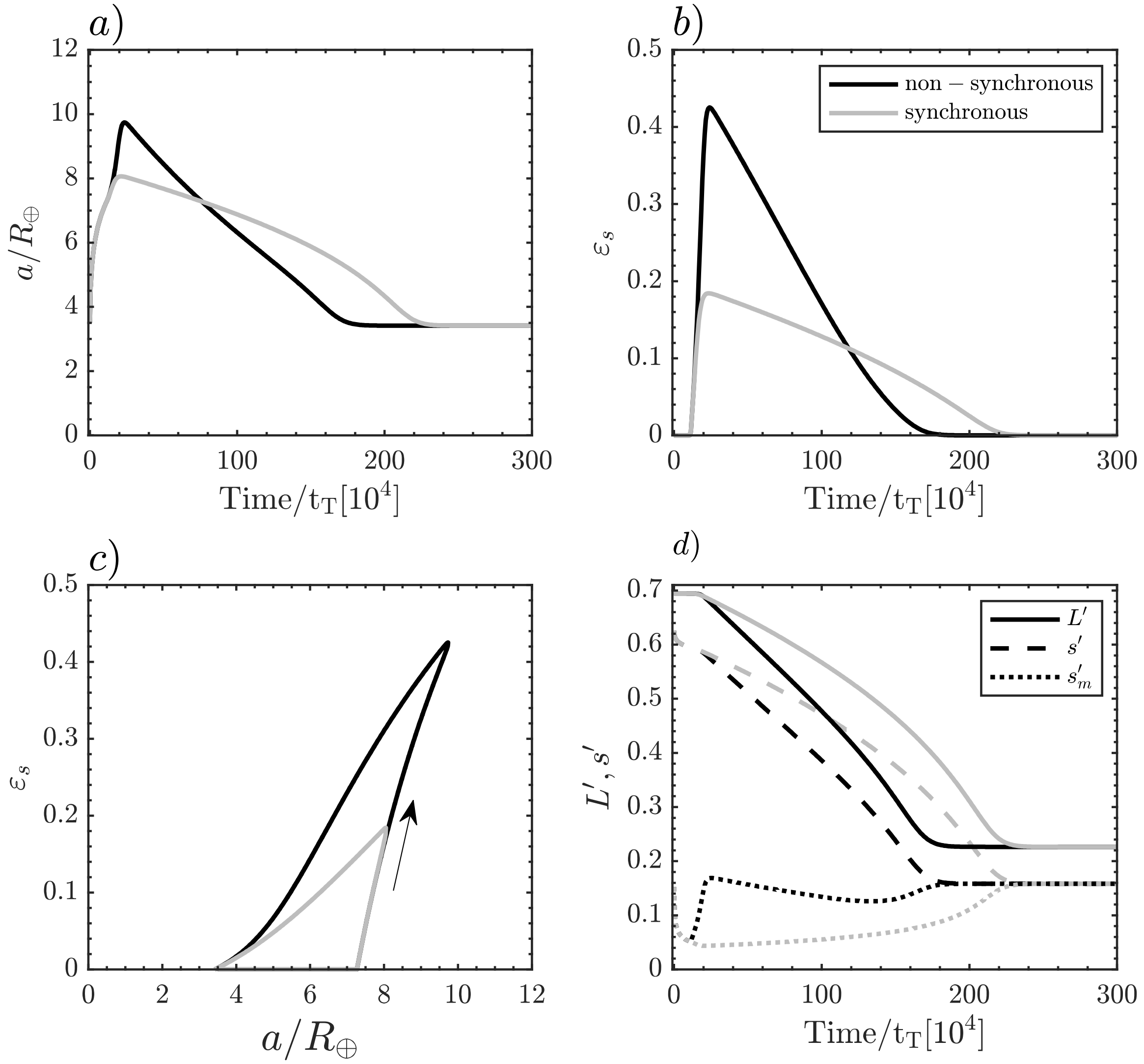

Fig. A3. System evolution for a Moon with synchronous rotation maintained by a permanent figure torque with $L_o = 2L_{EM}$ and $A = 10$ (grey), with non-synchronous rotation case from Figure 4 in the main text shown for comparison (black).
